# Fertility anxiety vs. anti-fertility anxiety: exploring Chinese women’s conflicting attitudes toward childbearing through social media

**DOI:** 10.3389/fpsyg.2025.1636612

**Published:** 2025-09-18

**Authors:** Weiwei Li, Yuchan Zhou

**Affiliations:** ^1^School of Philosophy and Social Development, Shandong University, Jinan, China; ^2^School of Journalism and Communication, Shandong University, Jinan, China

**Keywords:** fertility anxiety, anti-fertility anxiety, fertility attitudes, social media, mixed methods research

## Abstract

**Introduction:**

Amid a global decline in fertility rates, China’s consistently low birth rate has highlighted the importance of understanding the fertility attitudes of women of childbearing age. While previous studies have largely focused on fertility anxiety, less attention has been paid to anti-fertility anxiety—women’s psychological resistance to societal pressures and expectations surrounding childbearing. To address this research gap, this study explores how women construct and articulate these seemingly conflicting attitudes, as well as the sociopsychological mechanisms that enable their coexistence.

**Methods:**

Using grounded theory and natural language processing techniques, the study analyzed 4,495 Weibo posts (fertility anxiety = 2,761; anti-fertility anxiety = 1,734) through three-level coding, semantic network analysis, and sentiment analysis.

**Results:**

The findings indicate that both fertility anxiety and anti-fertility anxiety are shaped by sociocultural and media narratives at the macro level, family and workplace dynamics at the meso level, and personal values and experiences at the micro level. Fertility anxiety discourse is primarily situated at the macro and meso levels, reflecting women’s feelings of insecurity and powerlessness in the face of societal pressures, maternal penalties and unequal family burdens. Anti-fertility anxiety is more rooted in micro-level expression, emphasizing reproductive autonomy and identification with motherhood amid a broader feminist awakening. The emotional tendency of fertility anxiety is predominantly negative, with intensity decreasing from the macro to the meso and micro levels. Anti-fertility anxiety is associated with more positive emotions, with emotional intensity declining from the micro to the meso and macro levels.

**Conclusion:**

This study highlights the psychological tension between structural constraints and subjective desires in women’s fertility decisions, reinforcing explanations for conflicting fertility attitudes in the context of social media empowerment. These insights contribute to the conceptual understanding of anti-fertility anxiety and also offer practical recommendations for enhancing women’s reproductive autonomy, psychological well-being, and fertility-related policies and support systems.

## 1 Introduction

Global fertility rates have been in consistent decline, posing significant challenges to socioeconomic stability, including labor shortages, rising care burdens, and growing pressure on welfare systems (United Nations, 2024). China exemplifies this global trend. Since the implementation of the one-child policy in the late 1970s, the country’s fertility rate has fallen from 2.9 in 1979 to 1.41 by 2015 (Zeng and Hesketh, 2016; He et al., 2019). Despite the government’s relaxation of population control policies—first allowing two children per family in 2016 and then three in 2021 (Hong et al., 2022; Tatum, 2021)—these top-down interventions have failed to reverse the downward trajectory. In 2022, the total fertility rate reached a historic low of 1.08 (Zhai and Jin, 2023). This highlights the limitations of large-scale population policies in addressing low fertility rates and underscores the pressing need to investigate the micro-level psychological factors that influence individual fertility choices (Bongaarts, 2001).

Among these, women’s fertility attitudes are particularly central, as they shape both fertility intentions and outcomes (Khadivzadeh et al., 2018). Fertility attitudes encompass individuals’ cognitive and emotional orientations toward childbearing (Ajzen and Klobas, 2013). Empirical studies suggest that positive attitudes toward fertility often correlate with stronger intentions to have children (Mao and Luo, 2013). However, in contemporary China, these attitudes are increasingly characterized by anxiety. On the one hand, traditional gender norms—rooted in Confucian ideologies that equate womanhood with motherhood—continue to pressure women into childbearing roles (Zou and Wallis, 2021). On the other hand, modern women face structural barriers such as rising childbearing costs (Zhang, 2020; Chen et al., 2019), motherhood penalties (Han, 2025), and economic precarity (Qiao et al., 2024), resulting in what can be termed “fertility anxiety,” which refers to a state of internal conflict between normative expectations and perceived personal costs (Jiang et al., 2022; He et al., 2024a).

As they have become aware that this anxiety has increasingly shaped subjective well-being (Zhao et al., 2024) and decision-making (Jiang et al., 2022), some women have begun to re-evaluate their fertility anxiety, recognizing it as a condition worthy of resistance. Empowered by rising educational attainment (Gu, 2021), growing gender consciousness (Qian and Jin, 2018), increased labor market participation (Tong and Gong, 2020), and the spread of feminist discourse (Hao, 2024; Qiao et al., 2024), many women are reframing fertility anxiety as a socio-cultural construct rooted in institutional pressures. This shift has given rise to an emerging anti-fertility anxiety initiative—one that challenges moral and policy narratives linking fertility choices to national duty or feminine identity, and instead advocates for reproductive autonomy and the creation of a more supportive environment for childbearing.

Social media platforms have become crucial arenas where competing discourses around fertility anxiety and anti-fertility anxiety unfold. For instance, the Weibo topic “#Post-90s DINK couples being pressured by parents to have children” garnered over 25,000 comments and 300 million views, highlighting how deeply fertility anxiety resonates with younger generations caught in ongoing intergenerational conflicts over reproductive rights. Conversely, “#Freedom to procreate” attracted over 53,000 comments and 220 million views, indicating that those experiencing anti-fertility anxiety are actively advocating for reproductive autonomy. These digital discussions underscore the contested and evolving nature of women’s fertility attitudes.

Despite growing academic interest in fertility anxiety, significant research gaps remain. First, existing studies predominantly examine the antecedents and outcomes of fertility anxiety (Chan et al., 2020; Han, 2025; He et al., 2024a; Zhao et al., 2024), while paying insufficient attention to women’s agency in resisting these emotional pressures. Second, reliance on standardized survey instruments (Li et al., 2024; Qiao et al., 2024) has limited scholars’ ability to capture the rich, affective, and performative dimensions of fertility discourse, particularly as it unfolds in emotionally charged and interactive online environments (Zou and Wallis, 2021).

To fill these research gaps, this study conceptualizes “fertility anxiety” and “anti-fertility anxiety” as intertwined and seemingly contradictory dimensions of fertility attitudes, and further situates them within a media psychology framework. From the perspective of media psychology, media are regarded as crucial environments that shape individuals’ cognition, emotions, and behaviors (Fischer et al., 2011), particularly through psychological mechanisms such as social identity (Tajfel and Turner, 2004), social norms (Rodrigues et al., 2022), emotional contagion (Ferrara and Yang, 2015), and polarization (Iyengar et al., 2012). Social media are not only platforms for disseminating fertility-related information but also important psychological contexts where women experience intensified anxiety and engage in resistance through both cognition and emotion. Within this theoretical framework, fertility anxiety and anti-fertility anxiety can be understood as complex psychological effects jointly shaped by multiple mechanisms. Employing a mixed-methods approach, this study integrates grounded theory with natural language processing (NLP) techniques—including semantic network analysis and sentiment analysis—to examine how women construct and articulate reproductive attitudes on social media and uncover the socio-psychological processes behind these expressions. Four research questions are presented as follows:

1. What motivates the formation of fertility anxiety and anti-fertility anxiety among women on social media?
2. Which factors are most central in shaping these attitudes?
3. What emotional tendencies do women display when expressing fertility anxiety and anti-fertility anxiety?
4. How can these seemingly contradictory attitudes coexist within the same discursive context?

## 2 Literature review

### 2.1 Definition and causes of women’s fertility anxiety

The concept of anxiety has long been rooted in psychological research, typically linked to cognitive and emotional reactions to perceived threats or uncertainty (Miceli and Castelfranchi, 2005). However, recent studies have broadened this understanding, reframing anxiety as not merely an internal psychological state but also as a reaction to external social pressures. Scholars term this as “individual anxiety” or “social anxiety” (Hunt, 1999; Zhuang et al., 2022).

Within this framework, fertility anxiety has emerged as a significant subcategory. Gendered expectations around reproduction—shaped by both biological essentialism and social role theory—place the primary burden of reproductive responsibility on women (Chodorow, 2023). In this context, fertility anxiety refers to the emotional distress experienced by women of reproductive age when their subjective desire to have children comes into conflict with objective barriers to reproduction during the process of making reproductive decisions (He et al., 2024a).

Existing scholarship identifies three interrelated sources of fertility-related anxiety among women. First, concerns about the body and health serve as a fundamental source of anxiety. Many women report worries about both the physical and emotional demands of pregnancy, including fears surrounding fetal health (Campos et al., 2007) and postnatal bodily changes (Chan et al., 2020). Second, the socio-economic costs of childbearing generate significant pressure. Research on the “motherhood penalty” shows that women often face reduced work hours, higher risks of unemployment, and diminished income following childbirth (Killewald and García-Manglano, 2016; Wang et al., 2021). Beyond these financial impacts, additional burdens such as work–family conflicts (Li et al., 2024; Zhang and Tian, 2019) and the ongoing emotional labor of parenting (Jiang et al., 2022) further intensify anxiety. Third, cultural and familial expectations contribute to women’s fertility anxiety. Persistent intergenerational conflicts in parenting values (Zhang and Tian, 2019) and patriarchal norms such as son preference (Jiang et al., 2016) often create internal conflict and emotional strain, rendering reproduction a site of negotiation rather than personal autonomy (Logan et al., 2019).

Overall, prior studies have predominantly examined the negative causes and outcomes of fertility anxiety, while giving insufficient attention to how women perceive, navigate, and manage these anxieties, leaving a notable gap in understanding their subjective experiences.

### 2.2 The impact of feminist values on fertility attitudes

In recent years, feminist ideals emphasizing independence and self-actualization have gained growing momentum among Chinese women (Li, 2023). This expanding awareness has weakened the traditional link between marriage and fertility, granting women greater autonomy in these decisions (Hao, 2024; McQuillan et al., 2008) and reshaping their perceptions of motherhood.

At the individual level, digital feminist narratives champion women’s reproductive autonomy and challenge reductive views of women as childbearers, fostering acceptance of smaller and non-traditional families, especially among urban, educated women (Lu and Wu, 2024). Within families, feminist critiques oppose the gendered division of labor (Blaisure and Allen, 1995) and call for equal partnerships and shared domestic responsibilities to alleviate women’s caregiving burden (McRobbie, 2013). In the workplace, feminism advocates for gender equality through fair employment, pay equity, and family-friendly policies that enable women to pursue careers alongside reproductive choices (Saunders, 2016).

Contemporary feminist discourse on fertility does not reject motherhood but redefines it. Studies from contexts such as Hong Kong show that, despite declining birth rates, many women still desire motherhood. For them, motherhood represents not a concession to social expectations but an opportunity to foster emotional bonds and personal well-being (Adams, 2016). This reflects a feminist shift in the meaning of childbirth—from a state-imposed duty to a voluntary and meaningful life choice.

The aforementioned studies indicate that feminist values not only reshape societal understandings of reproduction but also provide critical resources and strategic frameworks for women coping with fertility-related anxiety. Building on this foundation, we introduce the core concept of anti-fertility anxiety and clarify its relationship to fertility anxiety. Anti-fertility anxiety refers to an emotional state in which women consciously resist and deconstruct traditional reproductive pressures when faced with fertility decisions and societal expectations. It is not merely the inverse of fertility anxiety but rather a critical emotional mechanism that differs in terms of subjectivity, cognition, emotion, and social function. Both anxieties often coexist in social media, sometimes within the same discourse. Indeed, fertility anxiety often catalyzes anti-fertility anxiety: when women recognize the constraints of fertility anxiety, they respond with acts of resistance and deconstruction. Fertility anxiety is characterized by repression and distress, whereas anti-fertility anxiety embodies critique and resistance—two states that are interrelated yet oppositional. The detailed comparison of the two can be seen in Table 1.

**Table 1:** The detailed comparison of fertility anxiety and anti-fertility anxiety.

Dimension	Sub-dimension	Fertility anxiety	Anti-fertility anxiety
Definition	—	Fertility anxiety is the emotional distress women of reproductive age feel when their desire to have children conflicts with actual barriers to reproduction while making reproductive choices (He et al., 2024a)	Anti-fertility anxiety refers to an emotional state where women consciously resist and deconstruct traditional reproductive pressures when facing fertility decisions and societal expectations
Difference	Subject differences	These are women who passively endure real-world constraints and external pressures	There are women who demonstrate critical awareness of reproductive challenges and actively articulate resistance
	Cognitive differences	It centers on the tension between reproductive desires and practical constraints, the discussion highlights concerns over economic, health, and career risks	It centers on the critique of reproductive norms and societal expectations, highlighting the redefinition of women’s reproductive rights and motherhood
	Emotional differences	It is characterized mainly by worry and helplessness, reflecting passive endurance of negative emotions	It is characterized mainly by healing and affirmation, often accompanied by positive dimensions such as liberation and self-empowerment
	Social function differences	It serves a reflective function, exposing the multiple pressures women face at the intersection of cultural norms, familial responsibilities, and personal aspirations	It embodies women’s critical awareness of reproductive norms, expressing their pursuit of autonomy and identity through psychological and social practices
Coexistence relationship	Causal relationship	It serves as the trigger, arising when women experience pressure from traditional reproductive norms, generating tension between societal expectations and personal aspirations	It represents the outcome, emerging as women recognize this tension and actively resist it
	Oppositional relationship	It reflects passivity, repression, and conformity	It embodies agency, resistance, and deconstruction

In conclusion, feminist values offer a vital theoretical framework for understanding and addressing fertility anxiety, while also contributing to the development of the concept of “anti-fertility anxiety.” However, there is a significant gap in systematic empirical research on how to effectively apply this framework within specific social contexts—particularly in highly interactive social media environments, where information and emotions flow rapidly. This analysis is essential for guiding women in reducing fertility anxiety and fostering more autonomous reproductive perspectives. Building on this foundation, we will explore reproductive discussions on social media to gain a deeper understanding of the dynamic tension between fertility anxiety and anti-fertility anxiety.

### 2.3 Fertility issues on social media

In the digital age, social media has become a vital space for public debate, emotional expression, and ideological negotiation around fertility (Deng et al., 2021). A large-scale study of over 240,000 posts from Chinese platforms identified three major themes: fertility trends, causes of reproductive challenges, and evaluations of fertility-support policies (Li et al., 2025). Notably, these online discussions often adopt a predominantly negative sentiment (Yang et al., 2024), contrasting with more positive narratives on Western platforms (Adair et al., 2014).

Two key mechanisms of digital platforms shape this negativity. First, social media amplifies perceived risks—ranging from health concerns, childcare burdens to career disruptions and environmental instability—heightening collective anxiety (Liu and Song, 2022). Second, emotional contagion spreads and intensifies these feelings as users mirror each other’s emotional expressions (Ye et al., 2024). Together, these dynamics foster a digital environment characterized by fear and uncertainty around fertility (Han, 2025).

However, the emotional landscape surrounding digital fertility issues is not entirely homogeneous, with social media increasingly evolving into a platform for resistance and reinterpretation. For instance, Chinese women’s childbirth vlogs and interactive comments sections create emotional communities where users assert reproductive agency and challenge traditional norms (Hua and Jiang, 2023). Similarly, during Italy’s #FertilityDay, women on Twitter redirected the conversation from state-driven natalism to broader issues of gender equity and maternal welfare policies (Micalizzi, 2021). These counter-discourses demonstrate that while social media can fuel anxiety, it also holds the potential for reimagining reproductive subjectivity and shaping public policy. Therefore, social media has become a critical platform for studying fertility and anti-fertility anxiety, which offers rich textual data that can complement traditional survey methods by overcoming their limitations in design and sample coverage.

In summary, existing research on women’s fertility anxiety has predominantly focused on its negative causes and consequences—including physiological, psychological, and broader social structural factors—while largely overlooking women’s expressions of anti-fertility anxiety shaped by feminist values. Traditional studies on fertility attitudes have also relied heavily on questionnaire-based methods, which are limited by fixed designs and restricted sample coverage, making it difficult to capture individuals’ dynamic emotions and social interactions in digital contexts. Social media, as a key site for emotional expression and value negotiation, has yet to be systematically examined in empirical research on the dual dimensions of fertility attitudes—anxiety and anti-fertility anxiety (Xie and Chao, 2022). To address these gaps, the present study examines fertility and anti-fertility anxiety on social media through analyses of thematic content, semantic networks, and emotional orientations. This approach reveals the tensions and resonances between these perspectives, offering a dialectical understanding of the diversity and agency in Chinese women’s fertility attitudes.

## 3 Methodology

**Figure 1:**
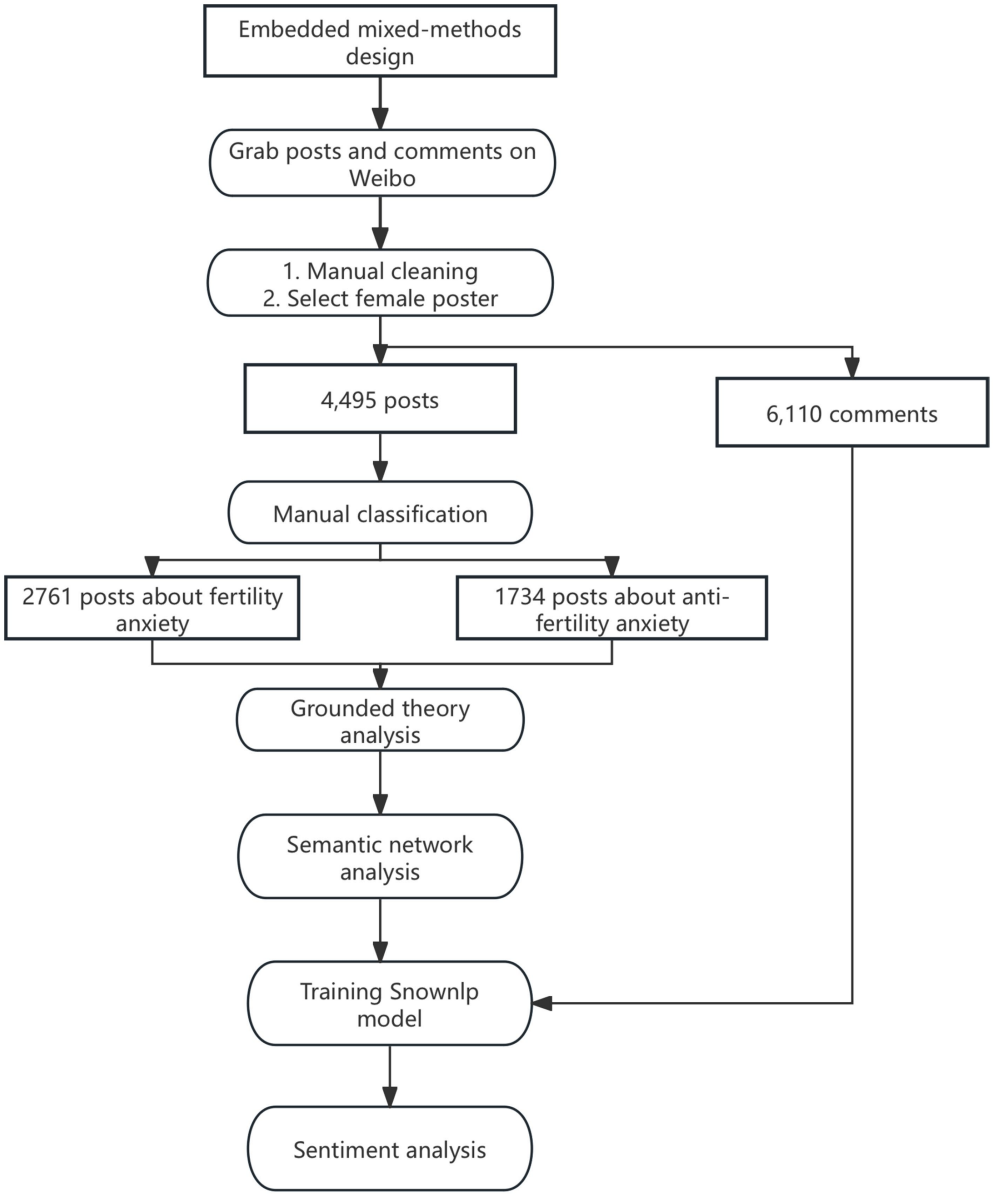
Embedded mixed-method research design.

This study employs an embedded mixed-methods research design, where the results from one method are used to expand, support, enrich, illustrate, and clarify the findings of the other (Sharma et al., 2023; Snelson, 2016). In accordance with the principles of embedded mixed-methods research, the study systematically integrates grounded theory and NLP. First, the grounded theory approach was used to identify thematic models related to fertility anxiety and anti-fertility anxiety on social media. Building on these qualitative insights, NLP techniques were applied to map the semantic networks of the themes and quantitatively analyze their emotional tendencies (see Figure 1). This integrated design enables a comprehensive exploration, in depth and scope, of how women articulate and negotiate these two fertility attitudes within China’s digital public sphere.

### 3.1 Data collection and cleaning

To achieve the research objectives, we used the software crawler Bazhuayu to collect original posts and associated comments containing the keyword “fertility” from Weibo between January 23 and 28, 2025. This period was selected because it was close to the traditional Spring Festival, a time when women of childbearing age often face heightened pressure from family and society to have children. Consequently, fertility-related attitudes tend to become more visible during this period. The dataset includes post content, URLs, timestamps, anonymized user profiles, and other metadata. All personally identifiable information, such as user IDs and contact details, was removed to ensure data privacy.

According to The British Psychological Society’s (2021) Ethical Guidelines for Internet-Mediated Research, the public nature of the Weibo platform implies that users posting publicly have a lower expectation of privacy regarding their views and opinions. Consequently, this study meets the criteria for waiving informed consent in accordance with these guidelines. Furthermore, all data collected from publicly accessible Weibo content have been fully anonymized, with all personally identifiable information—including user IDs and contact details—removed to ensure privacy and data security.

Data preprocessing was conducted in two stages. First, duplicate entries, advertisements, marketing content, and posts lacking sufficient contextual information were manually removed. Second, by integrating registration details, posted content, and community interactions, we inferred users’ gender and restricted the dataset to posts authored by female users. The final dataset consisted of 4,495 original posts and 6,110 comments.

Posts were manually categorized into two groups: fertility anxiety, which included expressions of fear, confusion, or internal conflict regarding childbearing, and anti-fertility anxiety, characterized by clear statements about whether or not to have children. Two trained research assistants independently coded all posts. Discrepancies were labeled as “uncertain” and subsequently resolved through discussion with a third coder. The final dataset comprised 2,761 posts under fertility anxiety and 1,734 posts under anti-fertility anxiety.

All Chinese materials were translated into English by bilingual researchers trained in the social sciences. The initial translation was carried out by the second author, while the first author identified key passages and conducted back-translation (Brislin, 1970) to verify both linguistic accuracy and conceptual consistency. Any uncertainties in the translation were addressed through regular team discussions.

### 3.2 Procedures

This study used NVivo 14 to conduct grounded theory analysis on two separate datasets to identify generative mechanisms within the qualitative data. Following a systematic process, we began with open coding, where two independent researchers analyzed the data line by line. For example, the phrase “not yet ready to accept the cost of fertility” was coded as “cost of fertility.” Similar expressions were grouped using NVivo’s node merging function. Coding discrepancies were resolved through discussion. After analyzing 500 posts from each dataset, Cohen’s Kappa coefficient increased from 0.68 to 0.83, indicating improved inter-coder reliability. Once open coding was complete, we conducted axial coding to explore relationships between the initial categories and identify overarching themes. In the selective coding phase, these categories were synthesized into a theoretical model explaining both fertility anxiety and anti-fertility anxiety. To assess theoretical saturation, 10 female reviewers with master’s degrees independently reviewed 20 posts from both categories, with 80% of reviewers agreeing that the model accurately captured women’s fertility attitudes, indicating theoretical saturation. This result also demonstrates the models’ strong explanatory power.

In the second phase, we constructed semantic networks to explore the central semantic structures of fertility anxiety and anti-fertility anxiety. The analysis involved the following key steps. First, we preprocessed the text using the Jieba word segmentation tool in the Python environment and eliminated stopwords with the extended Harbin Institute of Technology’s stopword dictionary. Next, we computed the word frequencies and applied Sun et al. (1999) dynamic high-frequency word formula to determine the dynamic threshold T, as follows:

$T=\sqrt{C\times D}$

where *C* is a constant adjustment coefficient and N is the total number of distinct words in the text. After iterative adjustments, we set *C* at 1.1 and extracted words with frequencies exceeding the threshold *T*. We then constructed co-occurrence pairs of high-frequency words, retaining only those with frequencies above a specified threshold of 8. Using the NetworkX library, we transformed these pairs into a semantic network, and visualized the network structure with the Spring Layout algorithm. Next, we evaluated node importance through degree centrality. Finally, the Louvain algorithm was applied to partition the network into communities, grouping semantically related words into clusters. We then further analyzed the co-occurrence patterns and semantic coherence within each community.

In the third phase, we performed sentiment analysis using SnowNLP, a Python-based tool for processing Chinese text. SnowNLP generates sentiment scores ranging from 0 (negative) to 1 (positive) using a Bayesian model trained on labeled data. However, as the tool’s default training corpus primarily consists of e-commerce reviews, which did not align with our research context, we created a custom sentiment corpus. Specifically, we manually labeled 5,607 comments as either positive or negative and used this labeled dataset to retrain the model by updating the pos.txt and neg.txt files. To validate the retrained model’s performance, we tested it on a separate set of 503 manually annotated comments, achieving an accuracy of 0.9065 (see Supplementary Figure S1), demonstrating the model’s reliability for our analysis. Next, we applied the sentiment analysis model to all posts and calculated sentiment scores for each sentence.

To evaluate emotional complexity, we calculated sentiment entropy, which is derived from Shannon’s information entropy formula and was applied by Quoidbach et al. (2014) to quantify emotional complexity. We used the following equation:


$$H\;(S)=-[p_{\mathrm{pos}\;\;}\cdot \log_{2}(p_{\mathrm{pos}})+p_{\mathrm{neg}\;\;}\cdot \log_{2}(p_{\mathrm{neg}})]$$

Where, Ppos denotes the probability or proportion of positive emotions, Pneg denotes the proportion of negative emotions, and H(S) ranges from 0 to 1. Higher entropy values reflect greater diversity in emotional expression, whereas lower values suggest more uniform or polarized emotional tendencies. This metric allows us to assess the breadth and complexity of sentiment expression within the data.

## 4 Results

### 4.1 Results of grounded theory coding

Grounded theory analysis of women’s social media discussions on childbearing reveals that “fertility anxiety” and “anti-fertility anxiety” form a dynamic system shaped by interactions across the micro, meso, and macro levels. While fertility anxiety is driven by structural pressures, familial and workplace inequalities, and individual fears and concerns, resistance strategies draw on personal agency and emotional ties, supportive family and workplace contexts, and broader social security measures. The following sections further unpack these two dimensions and their concrete manifestations.

#### 4.1.1 Coding results for fertility anxiety

**Figure 2:**
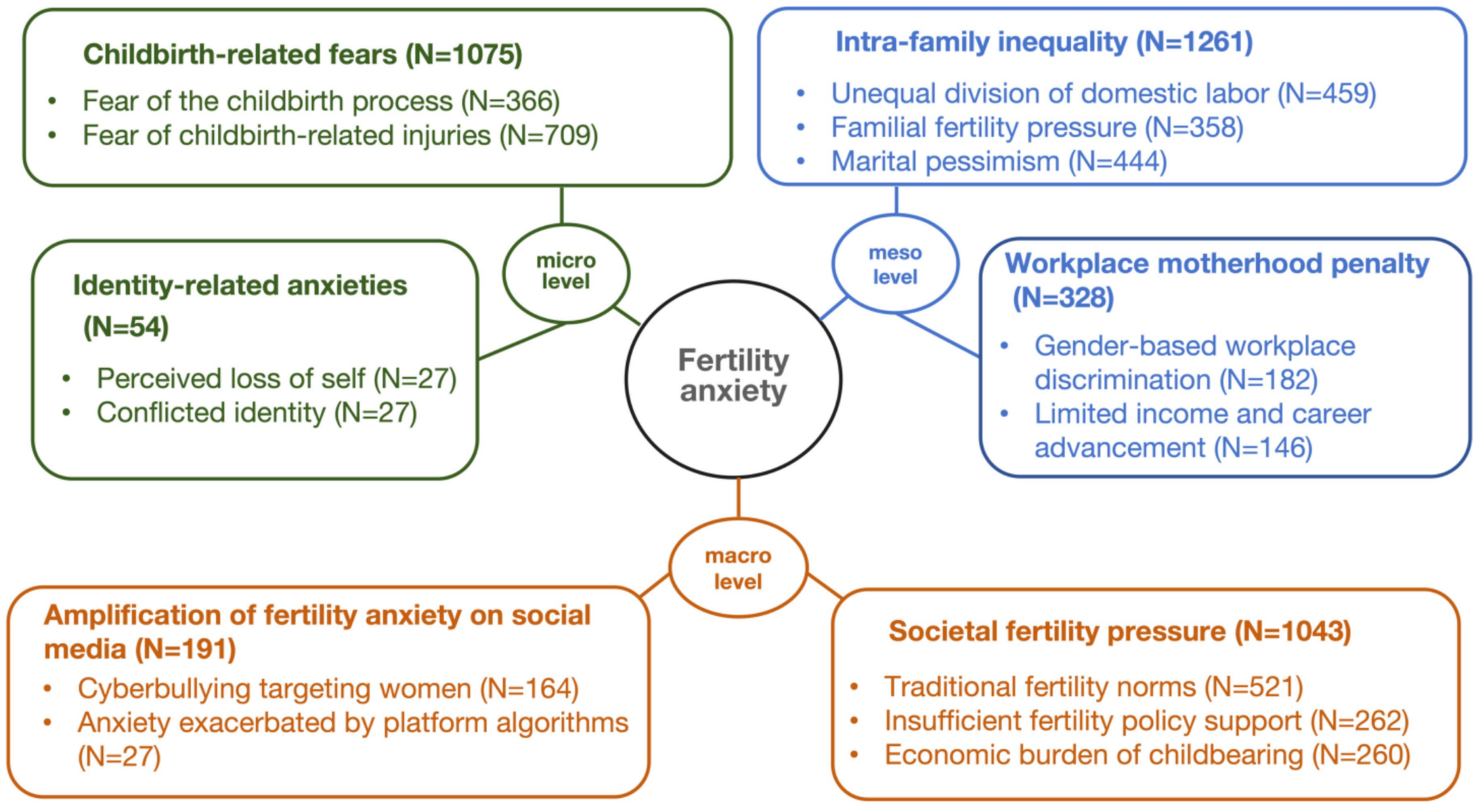
Theoretical model of the factors affecting women’s fertility anxiety. N represents the number of nodes.

Grounded theory coding reveals that women’s fertility anxiety arises from six main themes and 14 sub-themes, organized across three levels: macro, meso, and micro (see Figure 2). At the macro level, social pressure on fertility—intensified by the amplification of fertility anxiety on social media—creates widespread external forces that shape women’s fertility perceptions and decisions. At the meso level, inequality within families and the motherhood penalty in the workplace present concrete obstacles to childbearing. At the micro level, physiological fears and crises of identity further deepen women’s fertility-related anxiety. Details of the coding and reference sentences can be found in Supplementary data S1.

##### 4.1.1.1 Childbirth-related fears

A major driver of fertility anxiety is women’s psychological fear of childbirth, which centers on two main aspects. The first involves apprehension about the childbirth process itself, including potential risk and the intense pain of labor (Saisto and Halmesmäki, 2003). The second concerns childbirth-related injuries, such as stretch marks, weakened pelvic floor muscles, and urinary incontinence, along with medical issues such as postpartum depression and breast-related illnesses. Moreover, social anxieties about diminished sexual attractiveness and negative postnatal body image further compound these fears. For instance, under the hashtag “#The mom of twins with a hanging belly develops quilt-like creases after giving birth,” many female netizens reported that such physically harsh experiences intensified their anxiety surrounding childbirth.

##### 4.1.1.2 Identity-related anxieties

Beyond physical fears, many women experience a deeper crisis of identity linked to fertility. This anxiety arises from a perceived loss of self, as motherhood fragments their time, hinders career growth, and limits personal pursuits. The crisis is intensified by identity conflict, as women struggle to balance societal expectations of the “ideal mother” with other social roles (Haynes, 2006). The over-idealization of motherhood often suppresses or erases other dimensions of their identity.

##### 4.1.1.3 Intra-family inequality

Although families are typically viewed as sources of support, they can also contribute to anxiety through internal power inequalities (He et al., 2025). First, the division of domestic labor often burdens women with childcare responsibilities, limiting their autonomy and willingness to have children. Second, fertility pressure from family members, especially elders, frames childbearing as a filial duty. Third, pessimistic attitudes toward marriage—including distrust of intimate relationships and skepticism about the marriage contract—further intensify hesitation around childbearing.

##### 4.1.1.4 Workplace motherhood penalty

The motherhood penalty in the workplace—reflected in unequal pay, biased performance evaluations, and doubts about job suitability due to fertility (Correll et al., 2007)—heightens women’s anxiety. During recruitment, women frequently encounter gender-based discrimination, with employers probing into their marital status and reproductive plans. After becoming mothers, many are sidelined from important projects, denied promotions, or reassigned to marginal roles. In Chinese social media discussions, a typical case emerged when actress Jin Jing, upon returning to work after childbirth, was questioned by a male colleague about “why she was not staying at home to take care of her child.” This incident sparked widespread debate, and many female users pointed out that even public figures are not exempt from the motherhood penalty. These hidden penalties add emotional weight to fertility decisions. Moreover, the resulting income disparities and restricted career advancement force many women into a difficult choice between pursuing professional goals and starting a family.

##### 4.1.1.5 Societal fertility pressure

Fertility anxiety is deeply rooted in social pressures. First, traditional fertility norms idealize women as “virtuous wives” and “good mothers,” undermining their reproductive autonomy, while son preference further devalues their role. Second, insufficient fertility policy support—such as inadequate reproductive healthcare, limited childcare services, and weak protections against gender-based violence—intensify this burden by framing childbirth as a private issue rather than a public responsibility (Chen et al., 2022; Zeng, 2020). The case of a woman who endured 16 incidents of domestic violence over 2 years previously sparked broader discussions on Weibo concerning policies to safeguard women’s reproductive rights. Third, economic burden of childbearing, including high housing costs, education expenses, and job insecurity, also plays a major role. Despite China’s high female labor force participation at 60% in 2024 (World Bank, 2025), women remain economically vulnerable after childbirth, with customs such as bride price reinforcing insecurity.

##### 4.1.1.6 Amplification of fertility anxiety on social media

Social media amplification mechanisms exacerbate women’s fertility anxiety (He et al., 2024a). First, cyberbullying that targets women is rampant. In China, cases such as the “Fat Cat” incident have exposed widespread misogyny. In 2024, the incident began when Liu committed suicide amid a relationship dispute, sparking widespread online debate over alleged financial exploitation by his girlfriend Tan. However, investigations confirmed Liu’s gifts were voluntary and revealed his family orchestrated online harassment. This intensified gender tensions and triggered nationwide discussions on systemic gender inequality. This intensified gender tensions and triggered nationwide discussions on systemic gender inequality. Unmarried and childless women face moral judgment, while mothers are expected to meet perfectionist and often contradictory standards, leading to collective psychological harm. Second, platform algorithms exacerbate anxiety by portraying women as reproductive tools and disseminating idealized narratives about marriage and fertility. This creates an echo chamber effect, restricting diverse perspectives and narrowing women’s understanding of their reproductive choices.

#### 4.1.2 Coding results for anti-fertility anxiety

**Figure 3:**
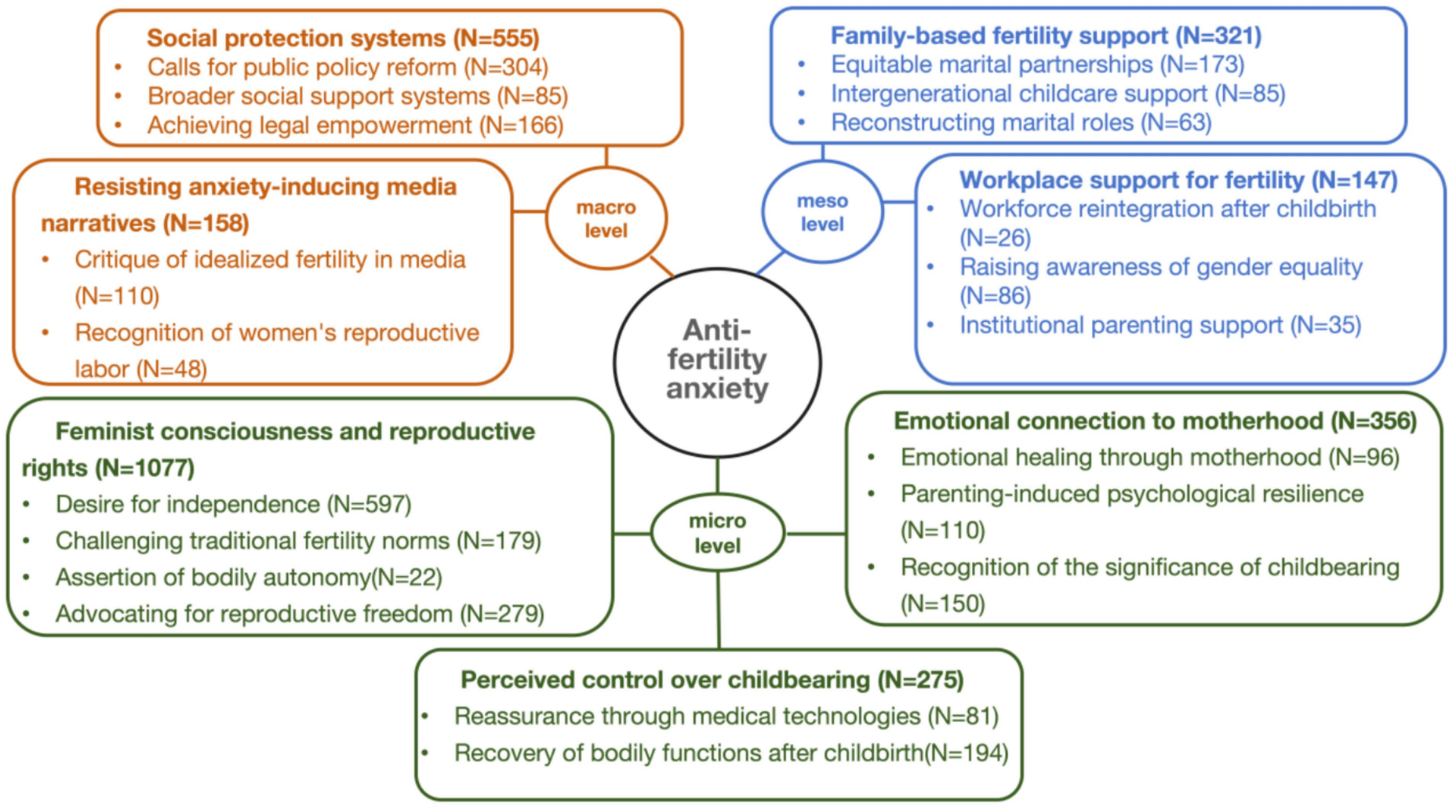
Theoretical model of the factors affecting women’s anti-fertility anxiety. N represents the number of nodes.

Based on grounded theory, women’s anti-fertility anxiety can be categorized into seven main themes and 20 sub-themes, organized across three levels: micro, meso, and macro (see Figure 3). At the micro level, a sense of control over childbearing, emotional identification with motherhood, and the defense of reproductive rights constitute internal coping mechanisms. At the meso level, supportive workplace and family environments help alleviate work–family conflict. At the macro level, improvements in social security systems and resistance to anxiety-inducing media narratives further alleviate reproductive anxiety. Together, these multi-level strategies form the core framework through which women resist fertility anxiety and lay the foundation for more autonomous, rational, and sustainable reproductive choices. Details of the coding and reference sentences can be found in Supplementary data S2.

##### 4.1.2.1 Perceived control over childbearing

Women view control over the childbirth process and its outcomes as a strategy for easing fertility-related fears. Medical technologies—such as painless delivery procedures and assisted reproductive methods—offer reassurance, particularly for those facing physiological challenges (Khademioore et al., 2023). Recovery of bodily functions, including pelvic floor and abdominal muscle rehabilitation, also helps restore confidence after childbirth.

##### 4.1.2.2 Emotional connection to motherhood

Emotional connection to motherhood can buffer against fertility anxiety (César et al., 2018). The emotional healing through motherhood, expressed through feelings of fulfillment, companionship, and emotional connection, provides a buffer against fertility anxiety. Children’s attachment and emotional feedback provide psychological sustenance, helping women transform anxiety into acceptance. Parenting also fosters resilience; a heightened sense of responsibility often encourages personal growth as women shift from passively accepting to actively embracing motherhood. Moreover, acknowledging the significance of childbearing not only highlights its essential role in sustaining life but also challenges idealized narratives of maternal sacrifice by promoting a more rational understanding of postpartum experiences.

##### 4.1.2.3 Feminist consciousness and reproductive rights

Women’s growing awareness of and advocacy for reproductive rights are central to addressing fertility anxiety. Driven by a desire for independence, they call for financial autonomy, reduced reliance on marriage, and equal access to education. They also challenge traditional fertility norms by promoting shared parenting, recognizing the value of domestic labor, and addressing the unequal burdens of motherhood (Han, 2025). Women assert their bodily autonomy by launching hashtag campaigns such as “#The Golden Age of Childbearing” to encourage others to reclaim control over their reproductive timelines and to challenge age-related collective anxiety. Furthermore, women advance reproductive freedom through legal reforms, such as rejecting restrictions on out-of-wedlock births, safeguarding breastfeeding rights, and promoting autonomy in reproductive decision-making.

##### 4.1.2.4 Family-based fertility support

Family support remains a critical buffer against fertility anxiety (He et al., 2025). Equitable marital partnerships, shared parenting duties, and emotional support during postpartum depression help reduce mothers’ sense of isolation. Intergenerational caregiving also plays a vital role, with grandmothers offering practical childcare support and emotional empathy. Additionally, women advocate for reconstructing marital roles that prioritize emotional intimacy over traditional procreative expectations, promoting autonomy and marital fulfillment.

##### 4.1.2.5 Workplace support for fertility

A supportive workplace is a crucial meso-level intervention. Workforce reintegration after childbirth strengthens women’s confidence in managing professional and parental roles (Chawla et al., 2024). Raising public awareness of workplace gender equality, combined with demands for equal parental leave and anti-discrimination policies, further reduces fertility-related anxiety. Institutional parenting support, such as managerial recognition of maternal competencies, peer support networks, and flexible work options, provides essential resources for working mothers.

##### 4.1.2.6 Social protection systems

Comprehensive social measures are essential for addressing fertility anxiety. Women are calling for public policy reforms, including extended paid maternity leave, universal childbirth allowances, and accessible childcare services, to redistribute the burden of childbearing from families to society. Broader social support systems—including full financial compensation for childbirth, eldercare provisions, dowry regulation, and assistance for vulnerable groups such as single mothers or stay-at-home mothers—are also needed. Furthermore, legal empowerment through measures against domestic violence, the elimination of marriage cooling-off periods, protection of inheritance rights, and penalties for surrogacy and human trafficking ensure that women’s reproductive rights are upheld.

##### 4.1.2.7 Resisting anxiety-inducing media narratives

Women are actively resisting anxiety-inducing media narratives through anti-fertility anxiety initiatives. They criticize media portrayals that idealize fertility, reinforce gender divisions, or dismiss women’s experiences. For instance, under the popular hashtag “#7-month-pregnant woman weighs only 104 pounds,” many female netizens voiced strong criticism of social media’s fixation on extremely thin pregnant women, contending that such portrayals reinforce unhealthy body standards. Additionally, they advocate for the recognition of women’s reproductive labor, emphasizing the need to acknowledge their contributions and promote reproductive freedom within safe and respectful environments.

### 4.2 Results of the semantic network analysis

This study further investigates the core dynamics of fertility anxiety and anti-fertility anxiety using semantic network analysis. The cluster results show that fertility anxiety is shaped mainly by intra-familial power imbalances, workplace penalties linked to motherhood, and systemic reproductive pressures at the societal level. In contrast, anti-fertility anxiety is rooted more in the micro level, reflected in women’s strong advocacy for reproductive autonomy within feminist frameworks and their positive emotional identification with maternal identity. The following subsections examine the semantic network features of each discourse in greater detail.

#### 4.2.1 Semantic network of fertility anxiety

**Figure 4:**
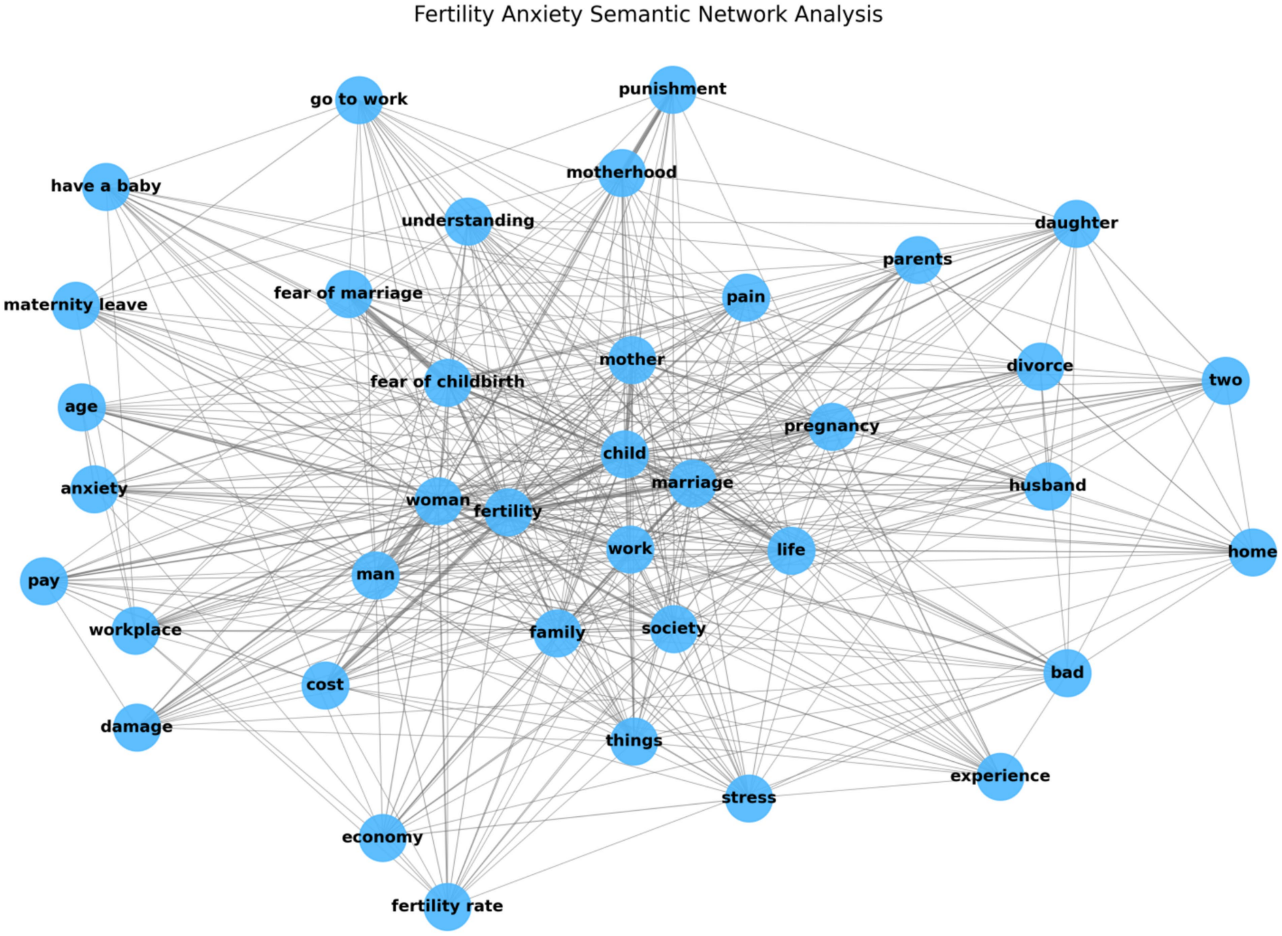
Semantic network of fertility anxiety.

To address Question 2, we conducted a semantic network analysis of fertility anxiety texts (see Figure 4). The network density was 0.6244, indicating strong overall connectivity. To gain a deeper understanding of the semantic structure, we performed a degree centrality analysis on the 38 nodes in the network (see Table 2). As is evident from the figure and table, nodes such as “fertility,” “marriage,” “child,” “woman,” “life,” “man,” and “mother” occupy central positions and exhibit high degree centrality. The strong interconnections—particularly between “fertility” and “child,” as well as between “fertility” and “marriage”—highlight their critical role in discussions of fertility anxiety. Nodes like “fear of marriage” and “fear of childbirth” directly reflect women’s attitudes toward childbearing. Additionally, nodes such as “stress,” “anxiety,” “pain,” and “bad” indirectly convey the instinctive feeling of fertility anxiety, establishing them as key emotional nodes in the network. Finally, nodes like “work,” “family,” “society,” “workplace,” “cost,” “economy,” “motherhood,” and “punishment” suggest that women’s fertility anxiety is significantly shaped by external structural factors.

**Table 2:** The central nodes of fertility anxiety.

Number	Nodes	Degree centrality	Number	Nodes	Degree centrality
1	Fertility	1	20	Workplace	0.5405
2	Marriage	1	21	Understanding	0.5405
3	Child	1	22	Parents	0.5405
4	Woman	1	23	Fear of marriage	0.5135
5	Life	0.973	24	Have a baby	0.5135
6	Man	0.9189	25	Stress	0.4865
7	Mother	0.8919	26	Cost	0.4865
8	Work	0.8919	27	Two	0.4865
9	Family	0.8649	28	Punishment	0.4865
10	Pregnancy	0.7568	29	Home	0.4595
11	Society	0.7297	30	Go to work	0.4595
12	Fear of childbirth	0.7027	31	Anxiety	0.4595
13	Husband	0.6486	32	Economy	0.4595
14	Bad	0.6216	33	Fertility rate	0.4324
15	Daughter	0.5946	34	Motherhood	0.4324
16	Things	0.5946	35	Pay	0.4324
17	Pain	0.5676	36	Age	0.4054
18	Divorce	0.5676	37	Maternity leave	0.4054
19	Experience	0.5676	38	Damage	0.3514

**Figure 5:**
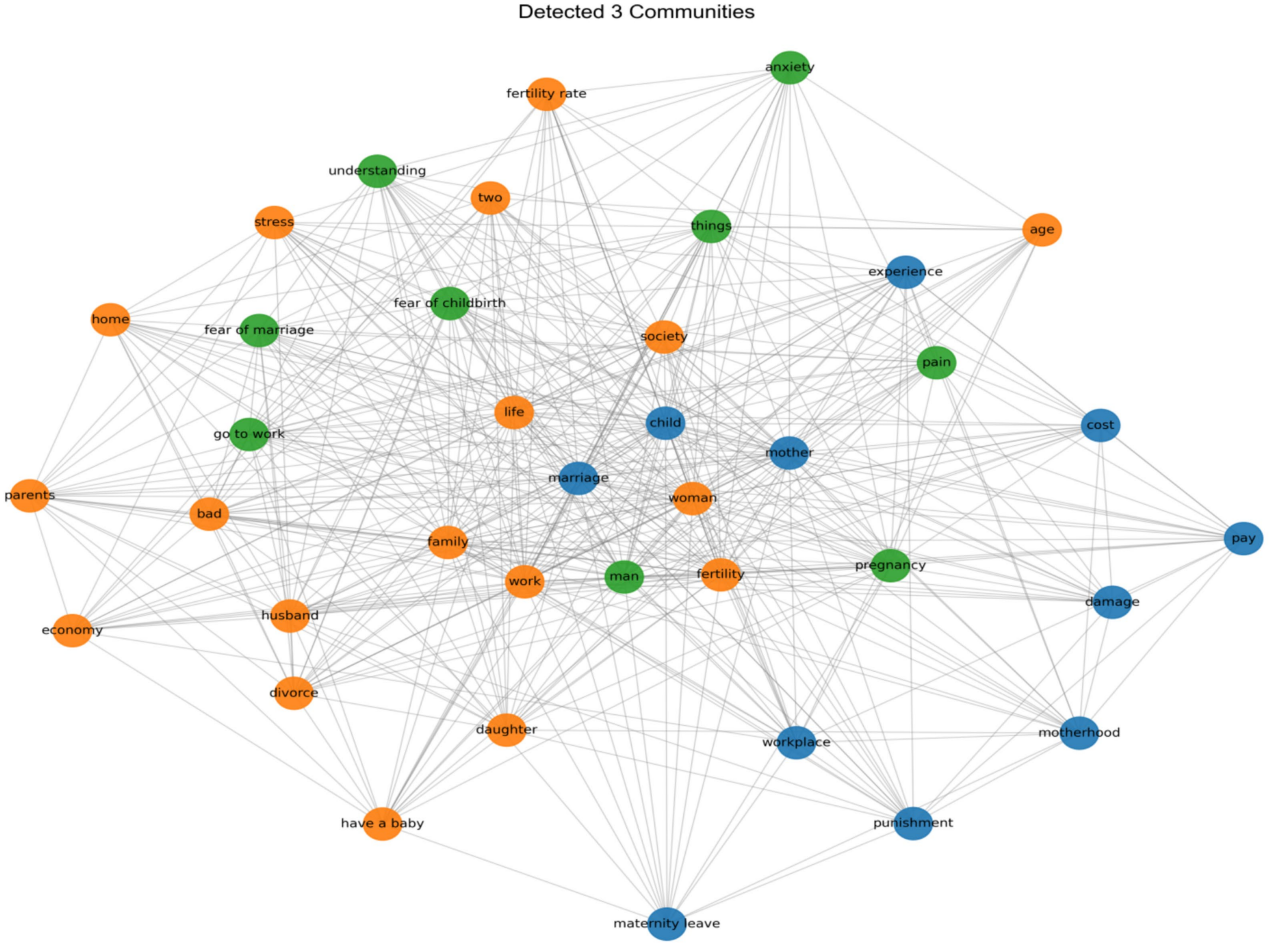
Detected communities in fertility anxiety text.

We further examined the network’s community structure (see Figure 5). The average clustering coefficient was 0.7746, indicating a strong community organization. Using a community detection algorithm, we identified three primary communities, each corresponding to a key aspect of women’s fertility anxiety.

Community 1 (Orange, 47.37%) highlights family and societal pressures relating to fertility. Terms such as “family,” “divorce,” “husband,” and “parents” reflect family-related pressures, particularly those arising from marital instability. Terms like “stress,” “society,” and “economy” emphasize the impact of economic conditions and social expectations on fertility anxiety.

Community 2 (Blue, 28.95%) focuses on workplace-related stress. Terms such as “motherhood,” “punishment,” “workplace,” and “cost” reveal the central role of motherhood-related penalties in fertility anxiety. Terms like “mother,” “marriage,” “child,” and “maternity leave” highlight the tension between career and childcare responsibilities, underscoring the challenges women face in balancing work and family life.

Community 3 (Green, 23.68%) is smaller and more dispersed, primarily reflecting women’s personal attitudes toward childbearing. Terms like “understanding,” “fear of marriage,” “fear of childbirth,” and “anxiety” reveal women’s emotional responses to fertility, particularly their fears and feelings of powerlessness regarding childbirth.

Overall, the results of these analyses validate the grounded theory coding, which asserts that women’s fertility anxiety is shaped by factors at the micro, meso, and macro levels. However, the semantic network analysis further refines this understanding by pinpointing the specific pressures women face in the family, workplace, and society. These pressures manifest as feelings of unease and fear. In line with grounded theory, anxiety at the family level is associated with domestic inequality, workplace anxiety is primarily driven by the motherhood penalty, and societal anxiety reflects systemic reproductive pressures. In summary, family inequality, the motherhood penalty, and societal fertility pressures are the key factors contributing to women’s fertility anxiety.

#### 4.2.2 Semantic network of anti-fertility anxiety

**Figure 6:**
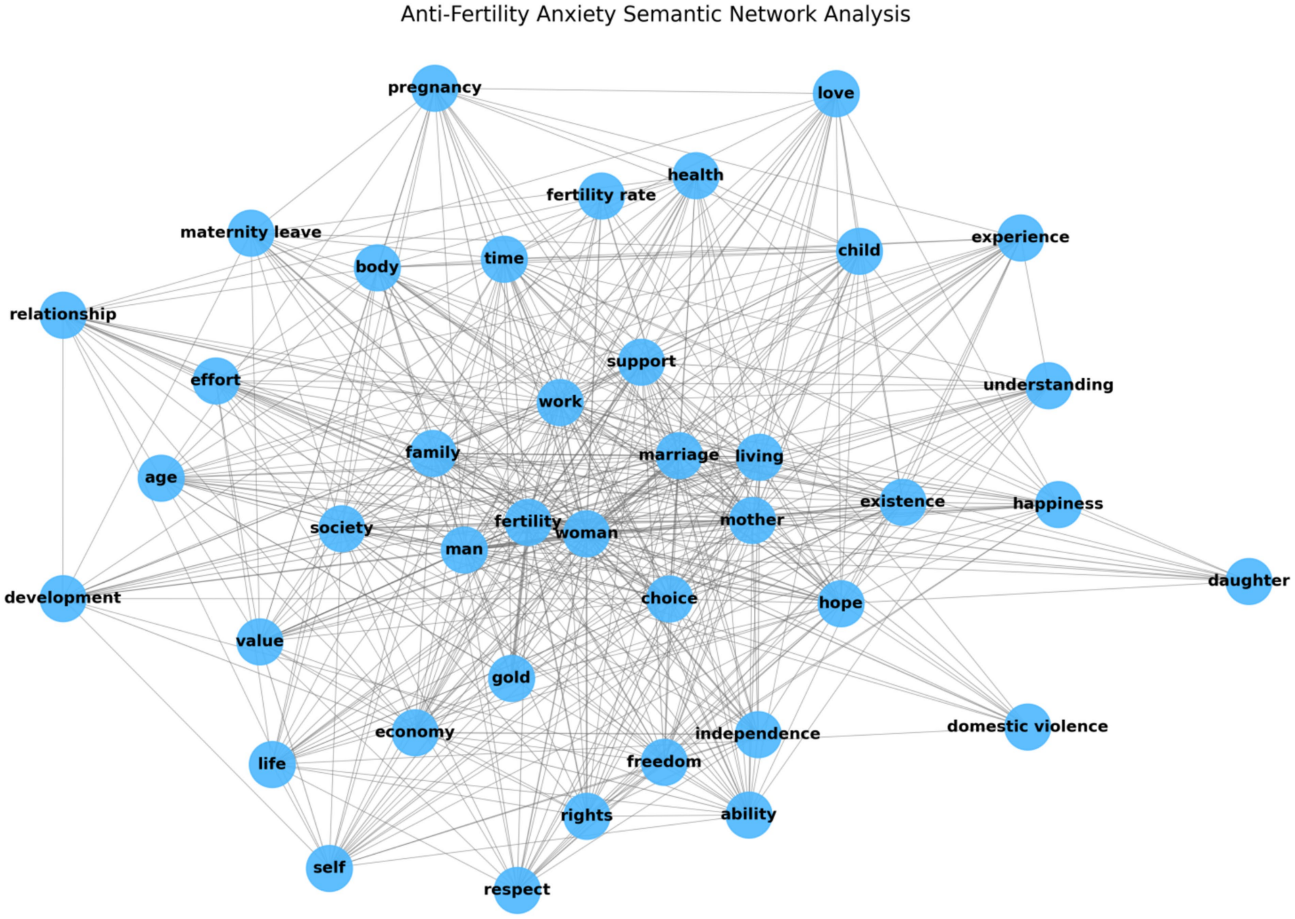
Semantic network of anti-fertility anxiety.

We conducted a semantic network analysis of texts related to anti-fertility anxiety (see Figure 6) and calculated a network density of 0.6064, indicating a high level of interconnectedness among the nodes. We then performed a degree centrality analysis on the 40 nodes in the network (see Table 3). Core nodes such as “fertility,” “woman,” “mother,” “marriage,” and “man” closely align with the central themes of fertility anxiety, reflecting women’s primary concerns about reproduction. Emotional support and psychological comfort are represented by nodes such as “happiness,” “hope,” “love,” and “understanding,” indicating that women seek emotional reassurance when making fertility decisions. Additionally, nodes such as “choice,” “rights,” “freedom,” “respect,” “health,” “value,” “economy,” and “independence” highlight women’s focus on autonomy and empowerment in the context of anti-fertility anxiety.

**Table 3:** The central nodes of anti-fertility anxiety.

Number	Codes	Degree centrality	Number	Codes	Degree centrality
1	Woman	1	21	Life	0.5897
2	Fertility	0.9744	22	Value	0.5385
3	Marriage	0.9744	23	Child	0.5385
4	Man	0.9487	24	Independence	0.5128
5	Mother	0.9231	25	Relationship	0.4872
6	Society	0.8718	26	Respect	0.4872
8	Choice	0.8462	28	Happiness	0.4872
9	Family	0.8205	29	Experience	0.4872
10	Hope	0.8205	30	Age	0.4615
11	Work	0.8205	31	Pregnancy	0.4615
12	Time	0.7692	32	Effort	0.4615
13	Existence	0.7179	33	Love	0.4615
14	Support	0.6667	34	Understanding	0.4359
15	Body	0.641	35	Maternity leave	0.4103
16	Economy	0.6154	36	Development	0.4103
17	Ability	0.6154	37	Gold	0.3077
18	Health	0.6154	38	Daughter	0.2564
19	Freedom	0.6154	39	Domestic violence	0.2564
20	Self	0.5897	40	Fertility rate	0.2051

**Figure 7:**
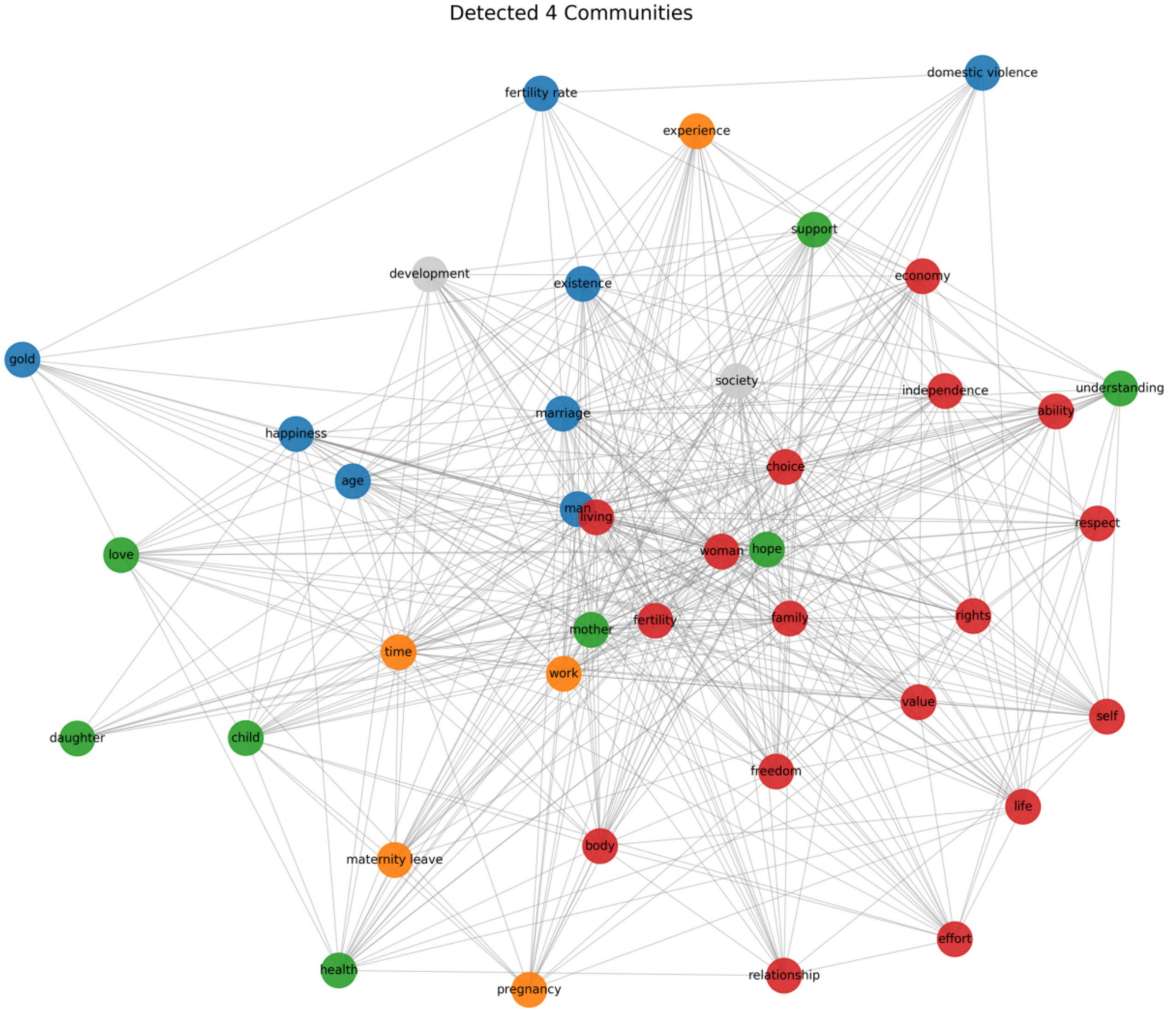
Detected communities in anti-fertility anxiety text.

The average clustering coefficient of the semantic network is 0.7651, indicating a robust community structure (see Figure 7). We identified four primary communities, each reflecting core aspects of women’s anti-fertility anxiety.

Community 1 (Red, 22.50%) focuses on women’s economic independence and reproductive autonomy. Terms such as “economic,” “independence,” and “ability” are directly linked to the pursuit of female autonomy within the context of feminist empowerment. Terms like “self,” “power,” “freedom,” and “choice” reflect women’s assertive stance in the fight for reproductive freedom and control over their bodies.

Community 2 (Green, 20.00%) addresses women’s emotional experiences related to motherhood. Terms like “mother,” “child,” and “daughter” highlight women’s evolving identities in their maternal roles. Terms such as “understanding,” “support,” “health,” and “love” underscore the emotional support and psychological fulfillment that motherhood provides.

Community 3 (Blue, 25.00%) centers on women’s challenges to traditional expectations regarding childbearing. Terms like “golden” (referring to the optimal age for childbearing) and “age” reflect women’s resistance to societal pressure about reproductive age. Additionally, “marriage” and “happiness” reflect shifting attitudes toward childbearing and marriage, while terms like “domestic violence” and “fertility” emphasize women’s concerns about reproductive rights.

Community 4 (Orange, 32.50%) highlights the influence of the workplace on women’s reproductive decisions. Terms such as “maternity leave” and “time” underscore the importance of workplace support. However, due to the relatively small number of nodes in this community, its overall impact appears limited, suggesting that workplace factors play a relatively minor role in fertility decision-making.

The analysis above suggests that women’s anti-fertility anxiety is predominantly rooted at the micro level. When integrated with the results from the grounded theory analysis, the terms in Community 1 and Community 3 underscore women’s advocacy for reproductive rights within a feminist framework. The terms in Community 2 highlight the emotional bonds related to motherhood, particularly the desire for support, understanding, and love, emphasizing the emotional resonance and comfort women derive from motherhood. Although support from family, society, and the workplace helps alleviate women’s fertility anxiety to some extent, these factors are relatively minor and dispersed within the overall network analysis. Thus, reproductive autonomy and emotional connections to motherhood emerge as the primary factors driving women’s anti-fertility anxiety.

### 4.3 Results of sentiment analysis

Overall sentiment analysis reveals a clear polarity between fertility anxiety and anti-fertility anxiety. Fertility anxiety is dominated by negative emotions, which gradually diminish from the macro to the micro level, whereas anti-fertility anxiety is characterized by positive emotions, decreasing from the micro to the macro level. This emotional distribution provides a foundation for understanding the internal affective structure of these discourses. Building on this, the following subsections present the specific emotional patterns of fertility anxiety and anti-fertility anxiety, illustrating their differentiated characteristics across levels.

#### 4.3.1 Sentiment patterns in fertility anxiety discourse

Using NLP techniques, we conducted a sentiment polarity analysis on discourses related to women’s fertility anxiety. The findings indicated a predominance of negative sentiment, with an overall sentiment score of 0.3521 (on a scale from 0 to 1). This is consistent with previous studies (He et al., 2024a). Negative sentiment accounted for 66.93%, while positive sentiment constituted only 33.07%. The sentiment entropy of 0.9117 suggests significant variability in emotional expression, reflecting the complexity of women’s fertility anxiety experiences.

An examination of emotional intensity across levels reveals a decreasing pattern from the macro (0.2822) to meso (0.3635), and micro (0.3959) dimensions in how women articulate their anxieties.

At the macro level, anxiety stemming from social media and societal pressures emerges as a significant contributing factor, although this level yielded the lowest sentiment score among the three (see Table 4). On social media, women expressed strong pessimism toward rampant cyberbullying, with 76.83% of related sentiments classified as negative. Harsh public commentary functioned as a societal barometer, deepening women’s sense of vulnerability and amplifying fears about fertility. Societal fertility pressure similarly scored low on sentiment (0.2850), underscoring a generally negative outlook. Notably, the low sentiment entropy values for insufficient policy support (0.7557) and the financial burden of childbearing (0.7588) reveal a shared emotional experience among women, highlighting persistent structural challenges.

**Table 4:** Sentiment score of fertility anxiety at the macro level.

Level	Core themes	Sub-themes	Score	Negative percentage	Positive percentage	Sentiment entropy
Macro level	–	–	0.2822	73.41%	26.59%	0.8355
	Societal fertility pressure	–	0.2850	73.18%	26.82%	0.8389
		Traditional fertility norms	0.3101	70.63%	29.37%	0.8734
		Insufficient fertility policy support	0.2587	78.24%	21.76%	0.7557
		Economic burden of childbearing	0.2590	78.08%	21.92%	0.7588
	Amplification of fertility anxiety on social media	–	0.2714	74.35%	25.65%	0.8214
		Cyberbullying targeting women	0.2435	76.83%	23.17%	0.7809
		Anxiety exacerbated by platform algorithms	0.4440	59.26%	40.74%	0.9751

At the meso level, the workplace motherhood penalty and intra-family inequality resulted in moderate sentiment scores (see Table 5). Among sub-themes, gender-based workplace discrimination against childbearing women recorded the lowest emotional score (0.2034) and the highest proportion of negative sentiment (83.52%), revealing enduring dissatisfaction with gender inequalities in professional settings. In the familial context, anxiety was evident in sentiments related to marriage (0.3092) and pressure to conceive (0.3400). These results illustrate how traditional norms continue to exert psychological pressure on women, significantly influencing their reproductive choices and contributing to a growing hesitancy toward childbearing.

**Table 5:** Sentiment score of fertility anxiety at the meso level.

Level	Core themes	Sub-themes	Score	Negative percentage	Positive percentage	Sentiment entropy
Meso level	–	–	0.3635	66.21%	33.79%	0.9228
	Intra-family inequality	–	0.3848	63.68%	36.32%	0.9453
		Unequal division of domestic labor	0.4931	52.07%	47.93%	0.9987
		Familial fertility pressure	0.3400	68.72%	31.28%	0.8964
		Marital pessimism	0.3092	71.62%	28.38%	0.8605
	Workplace motherhood penalty	–	0.2824	75.91%	24.09%	0.7965
		Gender-based workplace discrimination	0.2034	83.52%	16.48%	0.6458
		Limited income and career advancement	0.3807	66.44%	33.56%	0.9206

At the micro level, which encompasses anxiety related to childbirth and identity, women expressed relatively more positive emotions (see Table 6). Regarding physical health, fear of postpartum bodily harm (0.3773) surpassed fear of the childbirth process (0.4367). This disparity may be attributed to the broader availability of childbirth narratives that demystify delivery, whereas the potential for lasting postpartum damage evokes deeper anxiety. Women exhibit greater concern over identity than over physical fears, which suggests that the tension between preserving an independent identity and conforming to traditional maternal roles significantly amplifies fertility-related psychological distress.

**Table 6:** Sentiment score of fertility anxiety at the micro level.

Level	Core themes	Sub-themes	Score	Negative percentage	Positive percentage	Sentiment entropy
Micro level	–	–	0.3959	62.36%	37.64%	0.9554
	Childbirth-related fears	–	0.3972	62.05%	37.95%	0.9577
		Fear of the childbirth process	0.4367	58.47%	41.53%	0.9792
		Fear of childbirth-related injuries	0.3773	63.89%	36.11%	0.9435
	Identity-related anxieties	–	0.3703	68.52%	31.48%	0.8986
		Perceived loss of self	0.2981	77.78%	22.22%	0.7642
		Conflicted identity	0.4425	59.26%	40.74%	0.9751

#### 4.3.2 Sentiment patterns in anti-fertility anxiety discourse

Sentiment polarity analysis of discourse on anti-fertility anxiety reveals a predominantly positive emotional tendency. The overall sentiment score reached 0.6175, with 62.37% of expressions conveying positive emotions and 37.63% negative expressions. However, a high sentiment entropy value (0.9553) indicates significant emotional complexity within this discourse.

In contrast to fertility anxiety, sentiment scores for anti-fertility anxiety followed an inverse pattern, with positivity diminishing from the micro (0.6898) to meso (0.5393) and macro (0.4956) levels.

At the micro level, sentiment was highest, reflecting generally positive attitudes (see Table 7). Emotional connection to motherhood scored significantly high (0.8058), with emotional healing through motherhood reaching 0.8824—the most significant positive force in mitigating fertility anxiety. In contrast, sentiment related to feminist consciousness and reproductive rights was positive (0.6592) but tempered by emotional resistance to traditional norms. Perceived control over childbearing also scored highly (0.5585), reaffirming positive sentiment. However, sentiment entropy surrounding medical technologies is significantly high (0.9990), revealing divided views likely driven by concerns over risks, costs, and side effects.

**Table 7:** Sentiment score of anti-fertility anxiety at the micro level.

Level	Core themes	Sub-themes	Score	Negative percentage	Positive percentage	Sentiment entropy
Micro level	–	–	0.6898	29.98%	70.02%	0.8810
	Perceived control over childbearing	–	0.5585	33.00%	67.00%	0.9148
		Reassurance via medical technologies	0.5113	48.15%	51.85%	0.9990
		Recovery of bodily functions after childbirth	0.7327	22.73%	77.27%	0.7732
	Emotional connection to motherhood	–	0.8058	17.13%	82.87%	0.6607
		Emotional healing through motherhood	0.8824	9.38%	90.63%	0.4488
		Parenting-induced psychological resilience	0.7895	18.18%	81.82%	0.6840
		Recognition of the significance of childbearing	0.7687	21.33%	78.67%	0.7478
	Feminist consciousness and reproductive rights	–	0.6674	32.59%	67.41%	0.9107
		Desire for independence	0.672	31.66%	68.34%	0.9006
		Challenging traditional fertility norms	0.5402	47.49%	52.51%	0.9982
		Assertion of bodily autonomy	0.7131	27.84%	72.16%	0.8532
		Advocating for reproductive freedom	0.7080	28.32%	71.68%	0.8597

At the meso level, sentiment becomes more polarized (see Table 8). Family-based fertility support remains relatively positive, with a sentiment score of 0.5896. Intergenerational childcare support stands out with the highest score (0.6651), underscoring its crucial role in facilitating and encouraging childbirth. However, sentiment entropy is high (0.9848) in discussions around equitable marital partnerships, indicating mixed and divided opinions. In contrast, attitudes toward workplace support for fertility are significantly more negative (0.4294), with particularly strong pessimism surrounding efforts to address workplace discrimination (0.3131), reflecting ongoing dissatisfaction and a lack of confidence in achieving equity in the workplace.

**Table 8:** Sentiment score of anti-fertility anxiety at the meso level.

Level	Core themes	Sub-themes	Score	Negative percentage	Positive percentage	Sentiment entropy
Meso level	–	–	0.5393	45.94%	54.06%	0.9952
	Family-based fertility support	–	0.5896	39.88%	60.12%	0.9702
		Equitable marital partnerships	0.5617	42.77%	57.23%	0.9848
		Intergenerational childcare support	0.6651	30.59%	69.41%	0.8884
		Reconstructing marital roles	0.5646	44.44%	55.56%	0.9911
	Workplace support for fertility	–	0.4294	59.18%	40.82%	0.9755
		Workforce reintegration after childbirth	0.6099	42.31%	57.69%	0.9829
		Raising awareness of gender equality	0.3131	69.77%	30.23%	0.8841
		Institutional parenting support	0.5811	45.71%	54.29%	0.9947

From a macro-level perspective, the sentiment index reveals a relatively negative trend (see Table 9), indicating women’s doubts about the effectiveness of social protection systems and social media in alleviating fertility anxiety. Notably, the sentiment score regarding resistance to social media-driven fertility anxiety is particularly low (0.4104), reflecting a critical stance toward online aggression and emotional manipulation. Regarding protection provided by the social system, women generally express a moderately optimistic outlook (0.5198). However, sentiment related to legal empowerment recorded the lowest score across all categories (0.2769), reflecting deep concerns and marked dissatisfaction with the lack of effective legal safeguards against domestic violence and marital rights violations.

**Table 9:** Sentiment score of anti-fertility anxiety at the macro level.

Level	Core themes	Sub-themes	Score	Negative percentage	Positive percentage	Sentiment entropy
Macro level	–	–	0.4956	50.49%	49.51%	0.9999
	Social protection systems	–	0.5198	48.11%	51.89%	0.9989
		Calls for public policy reform	0.6575	33.22%	66.78%	0.9171
		Broader social support systems	0.5018	54.12%	45.88%	0.9951
		Achieving legal empowerment	0.2769	72.29%	27.71%	0.8514
	Resisting anxiety-inducing media narratives	–	0.4104	58.86%	41.14%	0.9772
		Critique of idealized fertility in media	0.3605	64.55%	35.45%	0.9380
		Recognition of women’s reproductive labor	0.5249	45.83%	54.17%	0.9949

## 5 Conclusion and discussion

In response to the growing challenges of low fertility, this study investigates the social psychological mechanisms that influence how Chinese women form and express conflicting attitudes toward childbearing on social media. The findings indicate that fertility and anti-fertility anxiety are intertwined in terms of underlying motivations, central concerns, and emotional expressions, forming a complex cognitive and affective framework. This highlights the need to reconceptualize the tension between structural pressures on women’s reproductive decision-making and their autonomy within a digital context. The following section outlines the theoretical and practical implications of these findings.

### 5.1 Underlying motivations of fertility anxiety and anti-fertility anxiety

Using grounded theory coding, this study identifies the root causes of women’s fertility anxiety. At the micro level, anxiety arises from physiological fears of childbirth, including uncertainty about the childbirth process (Dencker et al., 2019) and the psychological burden of potential trauma (Chan et al., 2020). At the meso level, workplace “motherhood penalties” (Han, 2025) and unequal family dynamics intensify these anxieties. At the macro level, societal pressure (Zhang et al., 2022) and anxiety-inducing social media environments (He et al., 2024a) shape reproductive attitudes.

A key finding of this study is that the crisis of identity that women experience constitutes a significant source of fertility anxiety. This indicates that anxiety is not only driven by external constraints—such as economic, temporal, or caregiving demands—but also by deeper tensions related to women’s identity conflicts within the social structure. Specifically, the significant gap between women’s ideal selves and the socially constructed image of the “ideal mother” perpetuated by fertility norms and regulations emerges as a core source of emotional distress.

In contrast, our analysis of anti-fertility anxiety reveals how women actively respond to reproductive challenges. For example, at the micro level, women emphasize the importance of controlling the childbirth process, cultivating emotional connection to motherhood, and safeguarding reproductive rights. At the meso level, they demand equal treatment in both the workplace and the family. At the macro level, they call for institutional reforms, including stronger legal protections and inclusive, supportive policy frameworks. These statements suggest that Chinese women’s perspectives on childbearing are shaped by feminist discourse (Lu and Wu, 2024). However, unlike the second wave of feminism that harshly criticized motherhood (e.g., Firestone, 2015), these discourses do not signify a wholesale rejection of fertility. Instead, they reflect a vision of “empowered motherhood”—a model that integrates reproductive autonomy with the aspiration for a just and enabling reproductive environment.

A comparative analysis of the findings reveals that women exhibit contradictory attitudes toward fertility, which are reflected in two distinct responses. At the micro level, some women experience anxiety stemming from uncertainty and identity crises related to the childbearing process, while others adopt an individual perspective, emphasizing reproductive autonomy, redefining motherhood, and asserting self-agency to resist such pressures. These seemingly opposing responses also resonate across the meso (family and workplace) and macro levels (social structures, cultural norms, and media environments). We suggest that this psychological tension arises from individual differences in how women navigate the relationship between group identity expectations and personal self-identity. According to social identity theory and self-categorization theory, an individual’s self-concept encompasses both personal identity and group identity (Hogg, 2016). When the norms, values, or expectations of the social groups to which one belongs conflict with personal needs, psychological tension or an identity crisis may result (Erikson, 1968). Within this theoretical framework, women’s fertility anxiety reflects the dissonance between societal cultural norms surrounding the “ideal mother” and their own lived realities. Conversely, anti-fertility anxiety illustrates how women can restore psychological equilibrium in the face of such tension by actively redefining motherhood roles and asserting autonomy and self-agency. This finding indicates that, amid social and cultural transformation and the rise of gender consciousness, women’s attitudes toward childbearing are inherently dynamic (Lappegård et al., 2021). By introducing the concept of “anti-fertility anxiety,” this study not only moves beyond previous research that framed anxiety as passive endurance but also demonstrates how women actively reinterpret reproductive issues under institutional pressure. In doing so, it enriches our understanding of the sociopsychological mechanisms shaping women’s reproductive choices.

### 5.2 Core factors of fertility anxiety and anti-fertility anxiety

Semantic network analysis reveals the core issues of fertility and anti-fertility anxiety. Discussions on fertility anxiety typically address family-level inequalities, including marriage pessimism and fertility pressures within families, as well as maternal penalties in the workplace and conflicts between family and career. These concerns also extend to macro-level structural issues, such as traditional fertility norms, economic burdens, and insufficient fertility support policies. Together, these multi-level factors form a core framework that highlights the structural inequalities in the family, workplace, and society as the underlying causes of women’s fertility anxiety (Xue et al., 2024). This is a pattern consistent with the findings of Deng et al. (2021).

In contrast, the discourse structure surrounding anti-fertility anxiety is shaped by a value-oriented network centered on women’s autonomy. Discussions primarily emphasize micro-level experiences and individual reproductive rights, including pursuit of independence, advocacy for bodily freedom and reproductive autonomy, critical reflection on traditional fertility norms, psychological resilience developed through parenting, and re-evaluation of the meaning of childbearing. These discourses underscore women’s agency and capacity for self-construction in reproductive decision-making, as well as their personalized reproductive claims.

While grounded theory reveals the emotional tension between fertility and anti-fertility anxiety among women in the context of reproductive issues, semantic network analysis further clarifies the sources of this tension. Fertility anxiety primarily arises from external structural pressures at the familial and societal levels, whereas anti-fertility anxiety reflects women’s reproductive claims at the individual level. These opposing forces generate particularly acute tension within the Chinese context. On the one hand, traditional Confucian culture (Zou and Wallis, 2021) and state reproductive policies (Zhang et al., 2024) jointly construct an externally imposed reproductive logic, reinforcing the disciplinary function of reproductive responsibility for women. From the standpoint of perceived social norms, institutional pressures and dominant cultural expectations serve as the central psychological mechanisms that provoke fertility anxiety among women (Rodrigues et al., 2022; Peng et al., 2023). On the other hand, the penetration of neoliberal values has led women to increasingly assert their rights regarding gender equality while balancing family and career (McRobbie, 2013), often expressing their autonomy in online spaces (Wallis and Shen, 2018). Through disentangling themselves from external expectations, women regain autonomy over their reproductive choices, shifting from a sense of obligation— “I should have children” “—to empowered self-determination—” “I decide whether or not to have children” (Ryan and Deci, 2000). Consequently, the coexistence of structural pressures and individual autonomy on social media not only underscores the complex dilemmas faced by contemporary Chinese women in reproductive decision-making but also exposes the deeper tensions and cultural fractures embedded in the gender politics of fertility.

### 5.3 Emotional tendency of fertility anxiety and anti-fertility anxiety

Using NLP sentiment analysis technology, this study quantifies the sentiment patterns embedded in online discourse related to fertility anxiety and anti-fertility anxiety. The results indicate that fertility anxiety is closely associated with negative emotions, with the intensity of these emotions gradually diminishing from the macro level to the meso and micro levels. This trend aligns with prior findings on Chinese women’s fertility concerns in digital spaces (Han, 2025; Ye et al., 2024; He et al., 2024b). In contrast, anti-fertility anxiety is characterized by a distinct emotional structure, with positive emotions decreasing from the micro to the meso and macro levels. Unlike in Western contexts, where positive emotions often reflect a desire for childbearing (e.g., Adair et al., 2014), in the Chinese context, these emotions are more indicative of heightened awareness of individual rights and resistance to institutionalized reproductive norms. This emotional structure also reflects the core mechanism described by the theory of emotional polarization: on the issue of fertility, differing positions not only diverge cognitively but also generate emotional opposition (Iyengar et al., 2012).

The trajectory of emotional expression serves as additional evidence of the persistent conflict between structural constraints and women’s reproductive autonomy. At the macro and meso levels, institutional pressure and prevailing cultural expectations frequently elicit negative emotional responses such as fatigue, anxiety, and resignation, reflecting the psychological toll of societal judgment (Milman and Sternadori, 2024). However, some women respond with emotional assertiveness, actively affirming their reproductive autonomy through positive or self-empowering sentiments such as healing and strengthened identity. These affective responses are not merely personal but also politically meaningful emotional practices. Through digital platforms, women engage in critiques of dominant motherhood discourses and articulate aspirations for a more equitable and liberated reproductive future (Cai and Liu, 2024). These dual emotional dynamics underscore both the increasing agency of contemporary Chinese women in reproductive decision-making and the central role of affect in shaping public discourse and contesting reproductive norms.

### 5.4 The coexistence of fertility anxiety and anti-fertility anxiety: a sociopsychological mechanism

This study shows that women express conflicting attitudes toward fertility anxiety and anti-fertility anxiety on social media, reflecting the psychological tension between structural pressures and individual reproductive autonomy. When mainstream discourse, family expectations, and policy guidance collectively constitute a powerful “reproductive disciplinary mechanism,” women are subjected to persistent social evaluations and role expectations, particularly during critical life stages such as marriage and childbirth (Gu, 2021). However, with rising educational attainment, heightened gender consciousness, and increasing individualism, an increasing number of women are prioritizing self-actualization and bodily autonomy, resisting traditional expectations regarding marriage and reproduction (Lesthaeghe, 2011). This tension is not merely a psychological dilemma but represents a deeper conflict between institutional structures and female subjectivity in contemporary society. Social media platforms act both as amplifiers of this contradiction (Liu and Song, 2022) and as key arenas where women articulate, negotiate, and perform these tensions. On the one hand, their algorithmic logic and attention-driven mechanisms may inadvertently reinforce anxiety, transforming reproductive decisions into “platformized” emotional issues (Yao et al., 2025), thereby intensifying women’s sense of uncertainty and powerlessness. On the other hand, these platforms provide a space to challenge mainstream reproductive norms, enabling emotional expression and collective resonance. Furthermore, drawing on emotional contagion theory (Ferrara and Yang, 2015), emotions expressed by female users on social media—whether the negative sentiments characteristic of fertility anxiety or the positive feelings associated with anti-fertility anxiety—can propagate and interact within online communities. This dynamic shapes the emotional climate of public discourse and, in turn, reinforces or modulates the social expression of fertility attitudes at both macro and micro levels. Ultimately, the complexity of women’s fertility attitudes on digital platforms reflects the structural contradictions embedded in contemporary society and their digital manifestations.

### 5.5 Contributions

First, the theoretical contribution of this paper lies in expanding the dimensions of attitudes toward childbearing. Unlike previous studies that primarily focused on fertility anxiety and its associated pressures (He et al., 2024a; Zhang and Hao, 2024), this study systematically proposes and elaborates a unique psychological resistance orientation—anti-fertility anxiety. It reveals how women navigate trade-offs across multiple dimensions—including autonomy, career development versus family expectations, and societal norms—in their reproductive decision-making. Furthermore, this study defines fertility anxiety and anti-fertility anxiety as interrelated yet distinct analytical categories, thereby constructing a comprehensive, multilevel theoretical framework. This framework organically integrates macro-level sociocultural norms, meso-level family and workplace contexts, and micro-level personal values and self-construction. Within this framework, reproductive attitudes are positioned as dynamic, multifactorial, and interactive processes, bridging the interpretive gap between macro-level structures and micro-level individuals in fertility research.

Second, this study expands the analytical lens of fertility research by highlighting social media as a key space for the construction and expression of both fertility anxiety and anti-fertility anxiety. Whereas prior studies have primarily emphasized its role in amplifying fertility anxiety (Yao et al., 2025), our findings underscore its equally vital function in articulating anti-fertility anxiety. Social platforms operate as accessible digital public spheres, enabling women to voice anxieties, seek emotional support, and contest pro-natalist expectations. In doing so, individual reproductive struggles are reframed as shared political issues. By mobilizing digital affordances, women cultivate networks of resistant feminist discourse, sustained through emotional resonance and semantic interaction.

Third, the concept of anti-fertility anxiety advances feminist critiques of procreative ideology by shifting attention from macro-level institutional oppression to the psychological mechanisms of resistance at the individual level. Unlike traditional feminist scholarship that situates motherhood primarily within power structures of patriarchy and capitalism (Firestone, 2015; Glenn et al., 2021), this study emphasizes how resistance is also internalized through women’s values, emotions, and pursuit of autonomy. Moreover, by engaging with the contradictions of “Made-in-China Feminism” (Wu and Dong, 2019), the concept captures the unique psychological struggles of Chinese women caught between Confucian cultural expectations and neoliberal pressures, alternating between fertility and anti-fertility anxieties. In doing so, it enriches feminist frameworks of the reproductive discipline–resistance chain (Han, 2025) and demonstrates how, within digital media environments, women actively reconstruct autonomy and articulate subjectivity through emotional resistance.

Fourth, at the methodological level, this study combines grounded theory with NLP, providing a new approach for qualitative-quantitative integration research on social media texts. Grounded theory helps to extract core concepts and generate theories (Bowling, 2005) while semantic network analysis and sentiment analysis reveal the semantic structure and emotional distribution underlying women’s fertility attitudes. The combination of the two not only enhances the systematicity and explanatory power of the analysis but also provides a methodological paradigm for gender and fertility studies in the context of digital sociology.

### 5.6 Policy recommendations

Building on the multi-level analysis of women’s fertility anxiety and anti-fertility anxiety, this section proposes targeted policy recommendations to reduce fertility anxiety, enhance women’s reproductive autonomy and psychological well-being, and foster a more equitable and supportive childbirth environment.

At the micro level, efforts should focus on reshaping fertility narratives within women’s communities and promoting psychological empowerment. Educational initiatives providing accurate, evidence-based information about reproductive health can help reduce fear, dispel misconceptions, and give women a stronger sense of control. Encouraging the sharing of diverse—and especially positive—childbirth experiences on social media can challenge dominant fear-driven or idealized narratives. Online communities should be supported in facilitating fertility discussions centered on autonomy rather than obligation. In this way, fertility can be reframed not as a societal duty but as a personal expression of choice, identity, and dignity.

At the meso level, reforms to workplace and family structures are essential for creating supportive conditions for fertility. In the workplace, employers should implement inclusive maternity and parental leave policies, offer flexible or remote work options, and establish safeguards against discrimination to ensure reproductive decisions do not result in career setbacks. Within families, the unequal distribution of caregiving responsibilities must be addressed. Promoting shared parenting and encouraging men to take active roles in childcare are vital for easing the burden on mothers and fostering greater gender equality at home. These workplace and family-level changes should work in tandem to support women’s smooth reintegration into professional and social life after fertility.

At the macro level, policy efforts should focus on strengthening the state’s role in supporting fertility welfare and reshaping public discourse. This includes increasing investment in areas such as childcare subsidies, universal childcare services, and comprehensive maternal health insurance. Simultaneously, social media platforms—in collaboration with state regulators—should implement mechanisms to monitor fertility-related content, curbing the spread of extreme or sensationalized narratives. Instead, these platforms should be encouraged to promote balanced, respectful, and informative discussions that reflect the complexity of reproductive choices and affirm women’s right to make decisions about their own bodies.

### 5.7 Limitations and future research directions

Although the present study reveals important findings, it has several limitations. One limitation concerns the data source. The research primarily relies on Weibo, a popular and representative social media platform in China (Zou and Wallis, 2021). However, Weibo’s user base is diverse and may include fake accounts, bots, or extreme views, including radical feminism. Despite efforts to clean the data, fully representing women from various ages, social classes, and regions remains challenging. These data limitations may have influenced the findings. Future studies should explore women’s fertility attitudes on other platforms, such as Rednote, Douban, Zhihu, and TikTok, to capture a broader range of emotional expressions and discourse structures.

Another limitation is the static nature of the sentiment and semantic network analysis. This study uses fixed network structures to examine the emotional orientations, semantic associations, and communication paths of themes. However, fertility-related issues are time-sensitive and event-driven, shaped by factors such as policy changes, celebrity events, and fertility subsidy announcements. As a result, key themes and network structures may evolve. Future research could incorporate time-series analysis or evolutionary network methods to better capture the dynamic nature of these communications.

## Data Availability

The original contributions presented in the study are included in the article/Supplementary material, further inquiries can be directed to the corresponding author.
