# Supplementary material for: Fertility anxiety vs. anti-fertility anxiety: exploring Chinese women’s conflicting attitudes toward childbearing through social media
Source: Front Psychol. 2025 Sep 18;16:1636612. doi: 10.3389/fpsyg.2025.1636612 (PMC12488452; doi:10.3389/fpsyg.2025.1636612)
Supplement: Supplementary file 1 [file Table_1.docx]

Supplementary Material

# Supplementary Data

**Supplementary Data S1.** Node Coding and Example Sentences for Fertility Anxiety.

| **Code** | **Reference Content（Example）** |
| --- | --- |
| **Macro level** |  |
| Amplification of fertility anxiety on social media |  |
| Anxiety exacerbated by platform algorithms |  |
| Reduction of women to reproductive machines | *In real life, I rarely see a woman truly supported by her husband's family. If the family is poor, women are seen as breeding machines.* |
| Fertility idealized | *Stop idealizing the "barely-showing baby bump" standard to sugarcoat childbirth.* |
| Cyberbullying targeting women |  |
| Collective psychological violence | *When they want something from us—like babies—they praise women and our bodies. But when we’re no longer "useful," like choosing not to have children, the mask drops* |
| Misogyny | *I stepped into the elevator and saw a few colleagues with beautiful plums their sister had bought. I casually said, "Wow, those look nice—I kind of want some too." Then, one woman’s son loudly declared, "See? I told you only women like this stuff!" Everyone laughed, and the elevator felt cheerful, but to me, it just felt uncomfortable.* |
| Moral criticism of female behavior | *Sometimes I read comments that really make me realize how ridiculously high society's expectations for mothers are. Isn't that just another way of boxing women into impossible roles?* |
| Societal fertility pressure |  |
| Economic burden of childbearing |  |
| High cost of living | *The real issue is that education, housing, and healthcare have become so expensive that having kids feels like a luxury most young people simply can't afford.* |
| Unequal asset division in marriage | *Why does the bride have to go to the groom's house for the New Year after marriage? The bride price is laughably small. If the groom marries into the bride's family, we'll split the costs of raising the kids 50-50. I won’t complain.* |
| Insufficient fertility policy support |  |
| Inadequate childbirth support policies | *Paid parental leave should be something every worker has, no questions asked. When we don't stand up for women's rights, it just shows how badly the system is failing.* |
| Inadequate healthcare policies | *If I had been pregnant, I would've lost my health insurance this month, along with my maternity coverage.* |
| Pervasiveness of gender violence | *She suffered abuse 16 times, beaten so badly that she now has to wear a diaper for the rest of her life—and still, she can’t get a divorce?* |
| Traditional fertility norms |  |
| Son preference | *Since her first two children were girls, they said she could only keep the pregnancy if it was a boy.* |
| Depriving women of their reproductive autonomy | *A twisted sense of morality sees male exploitation as virtuous and female bodily autonomy as evil.* |
| Societal expectations of motherhood | *You want me to study to ease society’s employment pressure, and then give birth right after graduation? To be a good wife and mother?* |
| **Meso level** |  |
| Intra-family inequality |  |
| Family fertility pressure |  |
| Forced marriage and childbirth | *Why do some parents think a girl has to get married as soon as she stops going to school and kick her out like she's a burden or something?* |
| Intergenerational role expectation conflicts | *Some parents may be quite ordinary, yet they believe having children makes them special. This belief isn't based on reason; it's passed down through generations.* |
| Marital pessimism |  |
| Disappointment in marriage | *Many Chinese men still haven’t figured out what it really means to be a father. Half the time, when they're with their kids, they’re just glued to their phones, which isn’t parenting.* |
| Hesitation about intimate relationships | *As someone who's scared of marriage and having kids, I've realized that even getting close to someone feels overwhelming.* |
| Unequal division of domestic labor |  |
| Exploitation of women's labor by the family | *On average, Chinese women spend three times more time than men on caregiving at home.* |
| Family-imposed child-rearing norms for women | *In 6.5 hours, from 10 a.m. to 4:30 p.m., I haven’t had a moment to eat or even turn on my work computer—childcare has consumed all my time.* |
| Gender imbalance in contributions | *Do hardworking moms ever get the credit they deserve? After childbirth, they're met with endless unseen chores, no extra help, and even more expectations—just for being women.* |
| Workplace motherhood penalty |  |
| Gender-based workplace discrimination |  |
| Workplace discrimination against married and mothers | *When you're pregnant, they say you don't care about your job—some might even fire you. When you return after a smooth recovery, men might ask why you didn’t stay home longer with the baby.* |
| Emotional burden | *In the workplace, childless people worry about you having kids, while parents wonder when you’ll have more. Those with kids worry about you missing work for childcare.* |
| Limited income and career advancement |  |
| Obstacles to career development | *In reality, many women face unfair treatment when they return to work after having a baby—like being reassigned to a different role or having their salary cut, which really holds back their career growth.* |
| Obstacles to financial income | *Women's income decreases by 4% for each child they have. And men's incomes go up by 6% when they become fathers. That's the motherhood penalty versus the fatherhood reward.* |
| **Micro level** |  |
| Childbirth-related fears |  |
| Fear of childbirth-related injuries |  |
| Appearance anxiety | *Looking in the mirror has now become a mental test: hyperpigmentation, sagging tummies, alarming incisions, stretched-out skin. It's horrible. The cost of childbirth is really too great!* |
| Physical injury | *After giving birth, I no longer experienced menstrual cramps, but new symptoms appeared—like stretch marks, pelvic floor muscle weakness, and urinary incontinence. These symptoms started showing up about a week before delivery, similar to the early pregnancy symptoms I had.* |
| Fear of the childbirth process |  |
| Death anxiety | *I don't just have fertility anxiety. I have death anxiety.* |
| The intense pain of childbirth | *The delivery room was filled with women in pain, and my contractions grew stronger. The pain became unbearable, so I asked for an epidural, but the doctor said I had to be 3 cm dilated.* |
| Identity-related anxieties |  |
| Perceived loss of self |  |
| Draining of energy | *Right now, my biggest fear about having kids is that they'll completely trap me. There's just so much I want to do—so many things I want to learn, try, and places I want to go. I can barely keep up with everything I already want to experience.* |
| Restriction of individual progress | *Women often marry too young or without fully understanding it, then rush into motherhood before they’re emotionally ready, sacrificing their own growth for their partners.* |
| Conflicted identity |  |
| The conflict between "perfect mother" and other identities | *I'm so anxious. I'm the baby's mom—of course I care! Of course, I want to give them the best. But at the same time, I want to go back to work soon. I feel so far from being the "perfect mom."* |

**Supplementary Data S2.** Node Coding and Example Sentences for Anti-Fertility Anxiety.

| **Code** | **Reference Content（Example）** |
| --- | --- |
| **Macro level** |  |
| Resisting anxiety-inducing media narratives |  |
| Critique of idealized fertility in media |  |
| Criticizing the propaganda trend of gender antagonism | *In recent years, the online environment has been eager to spark all kinds of confrontations—male and female confrontations, workplace confrontations—often at the expense of science and objective facts.* |
| Narrative strategies against the glamourization of childbirth | *It’s frustrating to see two childbirth-related topics trending as hot topics... Is it really logical to penalize motherhood? Is it right to spend half your life having a baby?* |
| Social media creates unnecessary anxiety | *Is it interesting to click on a trending search about the best age for women to have children?* |
| Recognition of women's reproductivelabor |  |
| Call for social security for women | *To boost the birth rate, we must advocate for the legal rights of mothers and housewives and demand greater protections for women's safety—especially through the power of social media.* |
| Highlighting the sacrifices made by women | *Only women truly understand the toll childbirth takes on our bodies. Just because we choose to make these sacrifices doesn't mean anyone else has the right to speak for us.* |
| Social protection systems |  |
| Achieving legal empowerment |  |
| Criminalize domestic violence | *Domestic violence is no longer brushed off as a private family matter but is now prosecuted under intentional injury charges. Only then can we meaningfully discuss women's fertility intentions.* |
| Criminalize surrogacy | *Surrogacy is opposed, regardless of the prerequisites.* |
| Elimination of the cooling-off period in divorce | *On one hand, they say there is freedom of marriage; on the other hand, they impose a cooling-off period for divorce.* |
| Ensuring women's full inheritance rights | *#Woman Claims Unmarried Childbirth Led to Revocation of Village Collective Dividends# What are the reasons for unmarried childbirth? From the woman's perspective, it's unacceptable!* |
| Broader social support systems |  |
| Economic compensation for childbirth | *The woman's employer should bear the loss of income due to her absence during childbirth, while the man's employer should cover maternity leave pay, childbirth expenses, and related rewards.* |
| Assistance for vulnerable groups | *Strengthen the protection of the vulnerable party in a marriage (e.g. full-time mothers) and give full-time mothers the benefits they are entitled to.* |
| Calls for public policy reform |  |
| Extension of maternity and paternity leave | *#NPC Delegate Proposes 30-Day Paternity Leave for Male Employees# Support! This is also a form of protection for women.* |
| Improving the support system for childcare | *Some advocate for encouraging childbirth through improved social welfare, like free healthcare, education, and childcare, which address the financial concerns of raising children.* |
| **Meso level** |  |
| Family-based fertility support |  |
| Equitable marital partnerships |  |
| Balance of reproductive responsibilities | *Both men and women should share the challenges of childbirth and the responsibilities of raising children.* |
| Emotional support from a partner | *Postpartum depression can be distressing. Men should be aware of it, support their wives emotionally, and help create a stable, nurturing home environment.* |
| Intergenerational childcare support |  |
| Empathy for intergenerational parenting dilemmas | *I have no doubt about my mom's love for me—it's the same unconditional love I feel for my own child.* |
| Intergenerational parenting support | *For dual-income families, balancing work, life, and childcare is exhausting—nannies are costly and unreliable, so help from grandparents can be a true lifesaver.* |
| Reconstructing marital roles |  |
| Increased sense of autonomy in marriage | *If we had safety, freedom, and support, marriage and parenthood would be choices made purely out of love.* |
| Emotional connections in marriage over traditional expectations | *The ideal marriage is one where both partners have equivalent social standing, and their mutual admiration extends beyond mere physical attraction to include spiritual compatibility.* |
| Workplace support for fertility |  |
| Workforce reintegration after childbirth |  |
| Motherhood and career can coexist | *They're independent and thriving in their careers, surrounded by support and care. For them, the right to have children and the right to work have never been mutually exclusive.* |
| Rebuilding confidence in the workplace | *I felt the impact of the motherhood penalty, so I kept working on myself to make up for the career slowdown that came with having a child and to rebuild my confidence.* |
| Raising awareness of gender equality |  |
| Gender-equal parental leave | *Extending maternity leave alone can worsen workplace bias against women. Equal parental leave for both men and women helps reduce discrimination and encourages shared parenting responsibilities.* |
| Institutional parenting support |  |
| Legal protection of women's rights | *The company strictly follows labor laws and does not lay off women during their protected periods, which shows how important it is to choose an employer that truly cares.* |
| The care and support of their colleagues | *After giving birth, the care and support from my colleagues gradually eased my anxiety and filled me with warmth.* |
| **Micro level** |  |
| Emotional connection to motherhood |  |
| Emotional healing through motherhood |  |
| Gaining love through becoming a mother | *The price of a difficult childbirth is so high—maybe from the moment you want a baby, you are destined to face hardships. But every time you see your baby, you feel so happy, and it’s all worth it!* |
| Positive feelings of mothers | *Before, I thought marriage was true happiness, but now? Having my adorable daughter makes me feel so blessed. Every moment with her fills me with joy!* |
| Parenting-induced psychological resilience |  |
| Embracing the shift from singlehood to motherhood | *The role of a mother will grant me limitless strength... From confusion to determination to anticipation, we are preparing to welcome you.* |
| Getting the energy to grow | *I want to prove that, as women, the harm and sacrifices from childbirth are like aging—both uncontrollable and controllable. I honor my bravery and will continue to love myself in the days ahead.* |
| Recognition of the significance of childbearing |  |
| Creative continuity | *She has grown into someone more accomplished than you, enabling the high-quality continuation of your life.* |
| Rationalization and emotional adjustment to fertility | *The prerequisite for having children is that one must first demonstrate life's joy.* |
| Feminist consciousness and reproductive rights |  |
| Advocating for reproductive freedom |  |
| Support for non-marital births | *Having a child as a single woman is perfectly okay—bless this brave woman.* |
| Reproductive decision-making should be respected | *Every woman should decide for herself whether she wants children—it's a choice and a right that deserves respect.* |
| Maternity is a personal right, not a duty | *A woman's ability to have children is not an obligation, nor should it be viewed as a punishment; it is a precious right.* |
| Assertion of bodily autonomy |  |
| Overcoming anxiety about reproductive age | *#PrimeChildbearingAge is also prime time for career growth, self-discovery, and exploration—don’t let this socially constructed idea create unnecessary fertility anxiety.* |
| Overcoming body shame | *Gynecological issues, like period shame, are too often treated as taboo—linked to sexuality or impurity. It's time to end the stigma and approach them with understanding and respect.* |
| Challenging traditional fertility norms |  |
| Promote shared parenting | *My husband helps with diapers, bedtime, and play, so he truly sees how hard parenting is—which makes him appreciate both me and our little family even more.* |
| Rejecting the pressure of traditional motherhood | *We need to let moms be themselves and let go of the guilt they didn't sign up for. They deserve the space and courage to walk away from what wasn't theirs to carry in the first place.* |
| Rethinking the traditional gender division of labor | *The old norm—men work, women stay home—no longer holds.* |
| Desire for independence |  |
| Defending the right to education | *Through education, these girls from impoverished areas are no longer limited by their circumstances—they rise up, strong and resilient. This is the true power of education.* |
| Female independence and self-realization | *Wishing my 30-year-old self to be strong and vibrant, rooted firmly in the earth with a straight spine. May I always stay bright and radiant, keep reaching higher, and never give up.* |
| Freedom from dependence on marriage | *A healthy marriage is built on mutual respect and care, where both partners are willing to compromise for each other, rather than one relying entirely on the other.* |
| Untying self-worth from childbirth | *Women must prioritize themselves, love themselves more, protect their bodies, and never sacrifice their physical well-being to chase societal expectations.* |
| Perceived control over childbearing |  |
| Reassurance through medical technologies |  |
| Assistive medical technology | *After going through childbirth, I have to say—epidurals are honestly one of the greatest things modern medicine has given us. I feel so lucky to live in a time where we have access to that kind of care.* |
| Modern technology reduces fertility risks | *Just 50 days later, I've already forgotten the pain—epidurals are a lifesaver, even if they slow things down. Our baby held strong for 29 hours, their steady heartbeat my light through it all.* |
| Recovery of bodily functions after childbirth |  |
| Repair of postnatal injuries | *Her postpartum recovery is excellent; at first glance, it's hard to tell she's given birth, as she maintains her appearance exceptionally well.* |
| Rebuilding confidence | *I began postpartum recovery and training when my baby was four months old—it healed me physically and mentally, sparked my love for yoga, and restored my confidence.* |

## Supplementary Figure S1


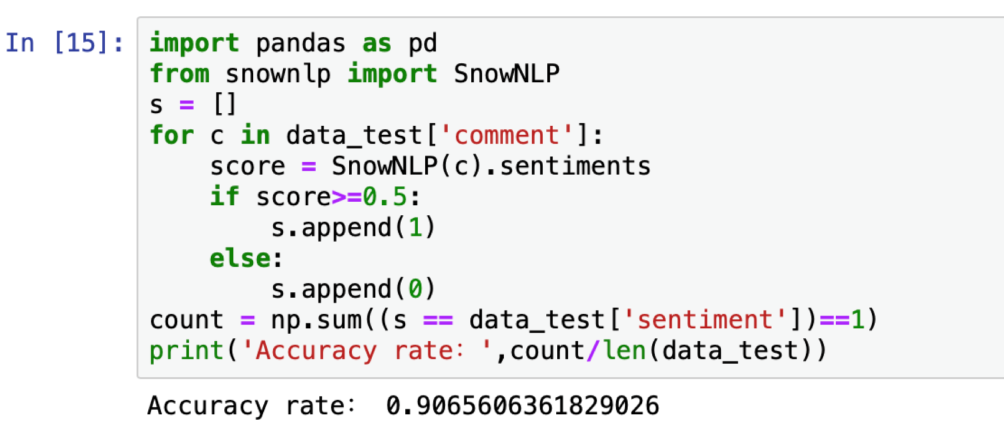


Figure S1. Training accuracy of the SnowNLP model
